# Millipedes and centipedes in German greenhouses (Myriapoda: Diplopoda, Chilopoda)

**DOI:** 10.3897/BDJ.2.e1066

**Published:** 2014-04-11

**Authors:** Peter Decker, Hans Simon Reip, Karin Voigtländer

**Affiliations:** † Senckenberg Museum für Naturkunde Görlitz, Görlitz, Germany

**Keywords:** Millipedes, centipedes, hothouse, Germany, first records, review

## Abstract

A review is given of all the literature records of millipedes and centipedes that have been found in German greenhouses together with additional records for 29 such sites. Species lists are given for 46 greenhouses investigated throughout Germany. Thirty-five diplopod and 18 chilopod species were found to occur in greenhouses, of which 15 (3 Chilopoda, 12 Diplopoda) are restricted to this type of habitat. First records for Germany include *Anadenobolus monilicornis* (Porat, 1876), Epinannolene cf. trinidadensis Chamberlin, 1918, *Epinannolene* sp., *Mesoiulus gridellii* Strasser, 1934, *Leptogoniulus sorornus* (Butler, 1876), *Rhinotus purpureus* (Pocock, 1894), *Cryptops doriae* Pocock, 1891, *Lamyctes coeculus* (Brölemann, 1889) and *Tygarrup javanicus* (Attems, 1907). The millipedes *Oxidus gracilis* (C. L. Koch, 1847) and *Amphitomeus attemsi* (Schubart, 1934) and the centipedes *Lithobius forficatus* (Linnaeus, 1758) and *Cryptops hortensis* (Donovan, 1810) are the species most frequently found in greenhouses.

## Introduction

Greenhouses provide different environmental conditions for animal colonization and survival compared with natural or synanthropic habitats outside. Greenhouses are characterized by a lack of frost and higher, usually constant temperatures, high humidity, permanent, regular watering, the presence of plants from mostly tropical countries and lack of or only low amounts of leaf litter and dead wood provide a more or less unique ecosystem inside such artificial buildings.

Examples of possible pathways of introduction of myriapods in the greenhouses can be found in Attems (1901) and Kraepelin (1901). Myriapods from all developmental stages (eggs, juveniles, adults) can be introduced easily within soil and other substrates associated with plants during shipment.

The apparent  first record of a myriapod species from greenhouses in Germany is that by Schnur (1857), who recorded "*Scolopendra germanica* C. L. Koch, 1837". This species should be referred to either *Cryptops hortensis* (Donovan, 1810) or *Cryptops parisi* Brölemann, 1920 (Attems 1930: 211). Which of these two species in fact was found by Schnur (1857) cannot be certain now today because the specimens have been lost. Another species recorded by Schnur (1857) is *Mecistocephalus maxillaris* (as *Geophilus maxillaris*). The identity of this species is doubtful and its specific status requires verification (Bonato and Minelli 2004). The same is true for the records by Latzel (1895) (*Mecistocephalus guildingii* Newport, 1844, later repeated as *Mecistocephalus maxillaris* by Jeekel (1964a)) and Eichler (1952).

**Figure 1.**
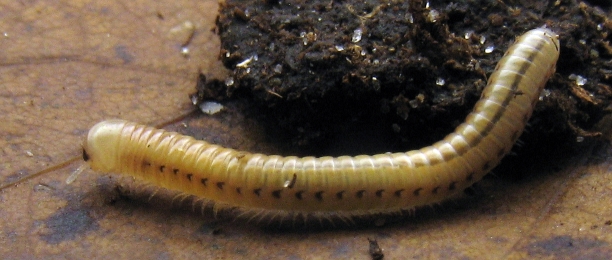
Living individual of *Paraspirobolus lucifugus* in the Hamburg Botanical Garden.

Latzel (1895) recorded *Oxidus gracilis* (as *Paradesmus gracilis*), *Mecistocephalus maxillaris*, *Lithobius forficatus* and *Paraspirobolus lucifugus* (as *Spirobolus dictyonotus* Latzel, 1885) (Fig. 1) from a hothouse in a nursery in Hamburg and *Poratia digitata* (as *Scytonotus digitatus* Porat, 1899) from a tannery near Hamburg. The record of *Paraspirobolus lucifugus* was wrongly referred to the tannery in Bergedorf near Hamburg by Schubart (1934).

Silvestri (1907) later described *Pectiniunguis pauperatus* Silvestri, 1907 from plant soil in the Hamburg Botanical Garden, but this species has never been found subsequently.

Reports of other species were published by Boettger (1929), Hahmann (1929a), Hahmann (1929b), Schubart (1925), Schubart (1929b), Schubart (1929a), Verhoeff (1891), Verhoeff (1907), Verhoeff (1934) and other authors. Most of these are summarized by Eichler (1952) together with his own observations in the Berlin-Dahlem Botanical Garden. In total this author listed 20 diplopod and nine chilopod species for Germany. In the following decades sparse records by Adis et al. (2000), Decker and Hannig (2011), Golovatch et al. (2001), Jeekel (1964a), Läffert (1984), Richter (1967), Schubart (1957), Thiele (1968), Wilck (2000) increased the number of species known from German greenhouses to a total of 23 diplopod and 12 chilopod species.

With regard to the other two myriapod groups, no Pauropoda are so far known from German greenhouses and for Symphyla the three species *Hanseniella oligomacrochaeta* Scheller, 2002, *Symphylella vulgaris* (Hansen, 1903) and *Hanseniella orientalis* (Hansen, 1903) were recorded from the greenhouses in the Berlin-Dahlem Botanical Garden (Scheller 2008). We did not carry out further investigationa for these two groups.

Records of millipedes and centipedes in greenhouses for most of other European countries are mostly scattered in the literature, e.g.: Austria (Golovatch and Sierwald 2001, Golovatch et al. 2001a, Golovatch et al. 2001, Gruber and Christian 2002, Gruber 2002), Belgium (Kime 2004), Denmark (Andersson et al. 2005, Blower and Rundle 1986, Enghoff 1975, Enghoff 1987), Czech Republic (Kocourek 2013), France (Brölemann 1896, Geoffroy and Iorio 2009, Gervais 1836, Golovatch and Sierwald 2001, Golovatch et al. 2001), Netherlands (Berg et al. 2008, Jeekel 1953), Poland (Jedryczkowski 1982, Jedryczkowski 1996), Slovakia (Mock 2001), Sweden (Andersson et al. 2005, Blower and Rundle 1986, Lohmander 1925, Porat 1889) and Switzerland (Bigler 1913, Holzapfel 1932, Pedroli-Christen 1993). Only the greenhouse fauna of Great Britain is quite well studied (e.g. Barber 1992, Barber 2005, Barber 2009, Blower 1985, Blower and Rundle 1980, Blower and Rundle 1986, Lee 2005a, Lee 2005b, Lee 2006, Lewis 2007, Pocock 1906, Read 2008) but lacks a comprehensive overview. A general overview of alien myriapods in Europe is provided by Stoev et al. (2010).

**Figure 2.**
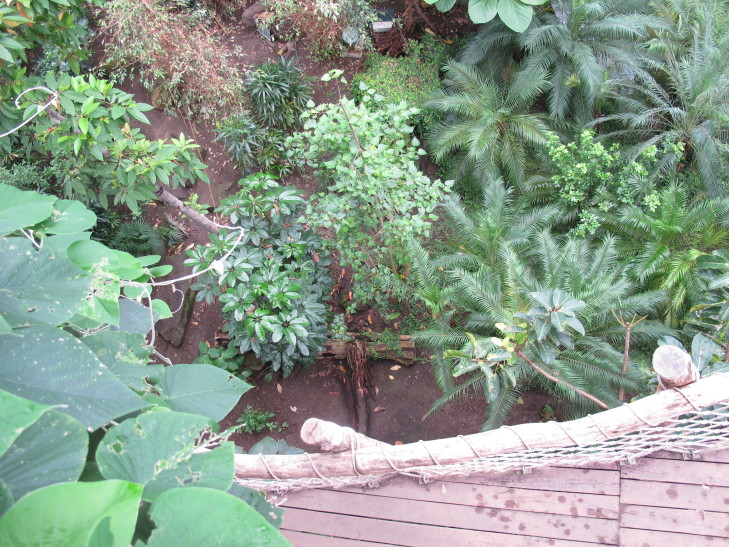
View from the top-of-the-tree-path in the greenhouse 'Gondwanaland', Zoological Garden Leipzig. Photograph: N. Lindner.

Currently, the increasing number of new constructions of tropical houses in Germany (Fig. 2) as pleasure grounds, butterfly houses or similar institutions has increased the number of exotic plants, mostly imported directly from tropical countries. The exchange of common plant stocks among botanical gardens has also supported the dispersal of alien myriapods.

## Materials and methods

In total, data for 46 greenhouses has been compiled (Table 1). Records from botanical gardens were not included if they had been made outside the greenhouses. Altogether we provide data for 53 species (35 Diplopoda and 18 Chilopoda) found in German greenhouses.

The present investigation is based on a comprehensive review of the literature and an analysis of the collections of the Senckenberg Museum of Natural History Görlitz (SMNG), the Museum of Natural History Berlin (ZMB), as well as of the data available in the Global Biodiversity Information Facility (GBIF, http://www.gbif.org, Edwards et al. 2000) and in the database on soil zoology, Edaphobase (http://www.edaphobase.org, Burkhardt et al. 2014).

In addition the myriapod faunas of 29 German greenhouses were investigated or reinvestigated by us (Table 1). For this part of our study Norman Lindner (Leipzig) provided highly valuable material from greenhouses in Berlin, Dresden, Konstanz, Leipzig and Potsdam. Altogether we collected more than 1800 specimens belonging to 41 species (29 Diplopoda, 12 Chilopoda). The sampling was done mainly by hand searching under stones or rotten plants and logs. Pitfall traps were used only in the Palm Garden in Frankfurt in 2008 by Heußler (2009), whose diploma thesis results are also included in the present study.

Material from these new samples has been mainly deposited in the Myriapoda collections of the Senckenberg Museum of Natural History Görlitz (SMNG). Record data is available online via the data portals of GBIF and Edaphobase. The full data sets with detailed information on site parameters, microhabitats and collection methods are available as Suppl. material 1. An overview of species and the cities is given in Suppl. material 2.

The natural distribution area is given for each species.

Nomenclature and classification follows Voigtländer et al. (2011).

## Checklists

### Checklist of centipedes and millipedes in German greenhouses

#### Chilopoda

Latreille, 1817

#### Lithobiomorpha

Pocock, 1895

#### Henicopidae

Pocock, 1901

#### Lamyctes coeculus

(Brölemann, 1889)

##### Materials

- **Type status:** Other material. **Occurrence:** recordedBy: N. Lindner; disposition: SMNG; **Location:** country: Germany; locality: Berlin; verbatimLocality: Berlin-Dahlem Botanical Garden; decimalLatitude: 52.4548; decimalLongitude: 13.3085; geodeticDatum: WGS84; **Event:** eventDate: March - April 2013
- **Type status:** Other material. **Occurrence:** recordedBy: N. Lindner; disposition: SMNG; **Location:** country: Germany; locality: Leipzig; verbatimLocality: Leipzig Botanical Garden; decimalLatitude: 51.3283; decimalLongitude: 12.3912; geodeticDatum: WGS84

##### Distribution

Southern hemisphere

#### Lithobiidae

Newport, 1844

#### Lithobius aeruginosus

L. Koch, 1862

##### Materials

- **Type status:** Other material. **Occurrence:** individualCount: 1; **Location:** country: Germany; locality: Kamen; verbatimLocality: hothouse near Kamen; decimalLatitude: 51.5900; decimalLongitude: 7.6600; geodeticDatum: WGS84; coordinateUncertaintyInMeters: 5800; **Event:** eventDate: 15 April 1905; **Record Level:** source: Verhoeff 1934

##### Distribution

Europe

#### Lithobius crassipes

L. Koch, 1862

##### Materials

- **Type status:** Other material. **Occurrence:** recordedBy: P. Decker & N. Laufer; individualCount: 1; disposition: SMNG; **Location:** country: Germany; locality: Darmstadt; verbatimLocality: Darmstadt Botanical Garden; decimalLatitude: 49.8700; decimalLongitude: 8.6798; geodeticDatum: WGS84; **Event:** eventDate: 20 January 2007
- **Type status:** Other material. **Occurrence:** recordedBy: P. Decker; individualCount: 1; disposition: SMNG; **Location:** country: Germany; locality: Mainz; verbatimLocality: Mainz Botanical Garden; decimalLatitude: 49.9925; decimalLongitude: 8.2454; geodeticDatum: WGS84; **Event:** eventDate: 04 November 2006

##### Distribution

Europe

#### Lithobius forficatus

(Linnaeus, 1758)

##### Materials

- **Type status:** Other material. **Occurrence:** recordedBy: J. Neubauer & K. Lebermann; individualCount: 3; disposition: SMNG; **Location:** country: Germany; locality: Bayreuth; verbatimLocality: Bayreuth Ecological Botanical Garden; decimalLatitude: 50.9810; decimalLongitude: 13.5730; geodeticDatum: WGS84; coordinateUncertaintyInMeters: 500; **Event:** eventDate: January - February 2012
- **Type status:** Other material. **Location:** country: Germany; locality: Berlin; verbatimLocality: Berlin-Dahlem Botanical Garden; decimalLatitude: 52.4548; decimalLongitude: 13.3085; geodeticDatum: WGS84; **Record Level:** source: Boettger 1929
- **Type status:** Other material. **Location:** country: Germany; locality: Berlin; verbatimLocality: greenhouses of horticulture company Bermann Rothe; decimalLatitude: 52.4320; decimalLongitude: 13.2580; geodeticDatum: WGS84; coordinateUncertaintyInMeters: 3000; **Record Level:** source: Boettger 1929
- **Type status:** Other material. **Occurrence:** recordedBy: P. Decker & N. Laufer; individualCount: 1; disposition: SMNG; **Location:** country: Germany; locality: Darmstadt; verbatimLocality: Darmstadt Botanical Garden; decimalLatitude: 49.8700; decimalLongitude: 8.6798; geodeticDatum: WGS84; **Event:** eventDate: 20 January 2007
- **Type status:** Other material. **Occurrence:** recordedBy: N. Lindner; individualCount: 1; disposition: SMNG; **Location:** country: Germany; locality: Dresden; verbatimLocality: Dresden Botanical Garden; decimalLatitude: 51.0435; decimalLongitude: 13.7582; geodeticDatum: WGS84; **Event:** eventDate: 10 March 2013
- **Type status:** Other material. **Location:** country: Germany; locality: Hamburg; verbatimLocality: greenhouses; decimalLatitude: 53.5500; decimalLongitude: 9.9900; geodeticDatum: WGS84; coordinateUncertaintyInMeters: 10000; **Record Level:** source: Latzel 1895
- **Type status:** Other material. **Occurrence:** recordedBy: N. Lindner; disposition: SMNG; **Location:** country: Germany; locality: Leipzig; verbatimLocality: nursery garden and horticulture Felgenträger; decimalLatitude: 51.3690; decimalLongitude: 12.4400; geodeticDatum: WGS84; **Event:** eventDate: 09 November 2008
- **Type status:** Other material. **Occurrence:** recordedBy: H. Reip; individualCount: 6; disposition: SMNG; **Location:** country: Germany; locality: Magdeburg; verbatimLocality: Gruson-Greenhouses; decimalLatitude: 52.1139; decimalLongitude: 11.6315; geodeticDatum: WGS84; **Event:** eventDate: 13 June 2012

##### Distribution

Europe

#### Lithobius lapidicola

Meinert, 1872

##### Materials

- **Type status:** Other material. **Location:** country: Germany; locality: Berlin; verbatimLocality: Berlin Old Botanical Garden; decimalLatitude: 52.4565; decimalLongitude: 13.3074; geodeticDatum: WGS84; **Record Level:** source: Eichler 1952

##### Distribution

Europe

#### Lithobius melanops

Newport, 1845

##### Materials

- **Type status:** Other material. **Occurrence:** recordedBy: P. Decker & M. Köhler; individualCount: 3; disposition: SMNG; **Location:** country: Germany; locality: Berlin; verbatimLocality: Berlin Zoological Garden; decimalLatitude: 52.5102; decimalLongitude: 13.3366; geodeticDatum: WGS84; **Event:** eventDate: 28 September 2013
- **Type status:** Other material. **Occurrence:** individualCount: 1; **Location:** country: Germany; locality: Kamen; verbatimLocality: hothouse near Kamen; decimalLatitude: 51.5900; decimalLongitude: 7.6600; geodeticDatum: WGS84; coordinateUncertaintyInMeters: 5800; **Event:** eventDate: 15 April 1905; **Record Level:** source: Verhoeff 1934
- **Type status:** Other material. **Occurrence:** recordedBy: N. Lindner; disposition: SMNG; **Location:** country: Germany; locality: Leipzig; verbatimLocality: Leipzig Botanical Garden; decimalLatitude: 51.3283; decimalLongitude: 12.3912; geodeticDatum: WGS84; **Event:** eventDate: December 1998
- **Type status:** Other material. **Occurrence:** recordedBy: N. Lindner; individualCount: 2; disposition: SMNG; **Location:** country: Germany; locality: Leipzig; verbatimLocality: nursery garden and horticulture Felgenträger; decimalLatitude: 51.3690; decimalLongitude: 12.4400; geodeticDatum: WGS84; **Event:** eventDate: 09 November 2008

##### Distribution

Europe

#### Lithobius microps

Meinert, 1868

##### Materials

- **Type status:** Other material. **Occurrence:** recordedBy: N. Lindner; disposition: SMNG; **Location:** country: Germany; locality: Berlin; verbatimLocality: Berlin-Dahlem Botanical Garden; decimalLatitude: 52.4548; decimalLongitude: 13.3085; geodeticDatum: WGS84; **Event:** eventDate: 15 May 2013
- **Type status:** Other material. **Occurrence:** individualCount: 1; **Location:** country: Germany; locality: Berlin; verbatimLocality: greenhouses of horticulture company Bermann Rothe; decimalLatitude: 52.4320; decimalLongitude: 13.2580; geodeticDatum: WGS84; coordinateUncertaintyInMeters: 3000; **Event:** eventDate: 15 June 1949; **Record Level:** source: Boettger 1929

##### Distribution

Europe

#### Lithobius mutabilis

L. Koch, 1862

##### Materials

- **Type status:** Other material. **Occurrence:** recordedBy: H. Reip; individualCount: 3; disposition: SMNG; **Location:** country: Germany; locality: Magdeburg; verbatimLocality: Gruson-Greenhouses; decimalLatitude: 52.1139; decimalLongitude: 11.6315; geodeticDatum: WGS84; **Event:** eventDate: 13 June 2012

##### Distribution

Europe

#### Scolopendromorpha

Pocock, 1895

#### Cryptoptidae

Kohlrausch, 1881

#### Cryptops hortensis

(Donovan, 1810)

##### Materials

- **Type status:** Other material. **Occurrence:** recordedBy: N. Lindner; individualCount: 2; disposition: SMNG; **Location:** country: Germany; locality: Konstanz; verbatimLocality: Mainau, butterfly house; decimalLatitude: 47.7068; decimalLongitude: 9.1951; geodeticDatum: WGS84; **Event:** eventDate: 21 April 2012
- **Type status:** Other material. **Occurrence:** recordedBy: H. Reip; individualCount: 2; disposition: SMNG; **Location:** country: Germany; locality: Magdeburg; verbatimLocality: Gruson-Greenhouses; decimalLatitude: 52.1139; decimalLongitude: 11.6315; geodeticDatum: WGS84; **Event:** eventDate: 13 June 2012

##### Distribution

Europe

#### Cryptops doriae

Pocock, 1891

##### Materials

- **Type status:** Other material. **Occurrence:** recordedBy: N. Lindner; individualCount: 3; disposition: SMNG; **Location:** country: Germany; locality: Potsdam; verbatimLocality: Biosphere Potsdam; decimalLatitude: 52.4185; decimalLongitude: 13.0486; geodeticDatum: WGS84; **Event:** eventDate: 19 August 2013

##### Distribution

Asia

#### Geophilomorpha

Pocock, 1895

#### Dignathodontidae

Cook, 1896

#### Henia vesuviana

(Newport, 1845)

##### Materials

- **Type status:** Other material. **Occurrence:** recordedBy: N. Lindner; individualCount: 2; disposition: SMNG; **Location:** country: Germany; locality: Dresden; verbatimLocality: Dresden Botanical Garden; decimalLatitude: 51.0435; decimalLongitude: 13.7582; geodeticDatum: WGS84; **Event:** eventDate: 10 March 2013
- **Type status:** Other material. **Occurrence:** recordedBy: N. Lindner; individualCount: 1; disposition: SMNG; **Location:** country: Germany; locality: Leipzig; verbatimLocality: Leipzig Botanical Garden; decimalLatitude: 51.3283; decimalLongitude: 12.3912; geodeticDatum: WGS84; **Event:** eventDate: December 1996

##### Distribution

Europe

#### Geophilidae

Leach, 1815

#### Geophilus flavus

(De Geer, 1778)

##### Materials

- **Type status:** Other material. **Occurrence:** individualCount: 1; **Location:** country: Germany; locality: Hamburg; verbatimLocality: Hamburg Botanical Garden; decimalLatitude: 53.5603; decimalLongitude: 9.9858; geodeticDatum: WGS84; **Event:** eventDate: 15 June 1949; **Record Level:** source: Jeekel 1964
- **Type status:** Other material. **Occurrence:** recordedBy: N. Lindner; individualCount: 2; disposition: SMNG; **Location:** country: Germany; locality: Leipzig; verbatimLocality: Leipzig Botanical Garden; decimalLatitude: 51.3283; decimalLongitude: 12.3912; geodeticDatum: WGS84; **Event:** eventDate: December 1999

##### Distribution

Europe

#### Geophilus electricus

(Linnaeus, 1758)

##### Materials

- **Type status:** Other material. **Location:** country: Germany; locality: Berlin; verbatimLocality: Berlin-Dahlem Botanical Garden; decimalLatitude: 52.4548; decimalLongitude: 13.3085; geodeticDatum: WGS84; **Record Level:** source: Eichler 1952

##### Distribution

Europe

#### Pachymerium ferrugineum

(C.L.Koch, 1835)

##### Materials

- **Type status:** Other material. **Occurrence:** recordedBy: H. Reip; individualCount: 4; disposition: SMNG; **Location:** country: Germany; locality: Magdeburg; verbatimLocality: Gruson-Greenhouses; decimalLatitude: 52.1139; decimalLongitude: 11.6315; geodeticDatum: WGS84; **Event:** eventDate: 13 June 2012

##### Distribution

Europe

#### Himantariidae

Bollman, 1893

#### Haplophilus subterraneus

(Shaw, 1794)

##### Materials

- **Type status:** Other material. **Occurrence:** individualCount: 1; **Location:** country: Germany; locality: Hamburg; verbatimLocality: Hamburg Botanical Garden; decimalLatitude: 53.5603; decimalLongitude: 9.9858; geodeticDatum: WGS84; **Event:** eventDate: 30 December 1950; **Record Level:** source: Jeekel 1964

##### Distribution

Europe

#### Mecistocephalidae

Bollman, 1893

#### Mecistocephalus maxillaris

(Gervais, 1837)

##### Materials

- **Type status:** Other material. **Location:** country: Germany; locality: Trier; verbatimLocality: surroundings of Trier; decimalLatitude: 49.7530; decimalLongitude: 6.6400; geodeticDatum: WGS84; coordinateUncertaintyInMeters: 5000; **Record Level:** source: Schnur 1857

##### Distribution

Asia

#### Tygarrup javanicus

(Attems, 1907)

##### Materials

- **Type status:** Other material. **Occurrence:** recordedBy: N. Lindner; individualCount: 6; disposition: SMNG; **Location:** country: Germany; locality: Berlin; verbatimLocality: Berlin-Dahlem Botanical Garden; decimalLatitude: 52.4548; decimalLongitude: 13.3085; geodeticDatum: WGS84; **Event:** eventDate: 15 May 2013
- **Type status:** Other material. **Occurrence:** recordedBy: N. Lindner; individualCount: 2; disposition: SMNG; **Location:** country: Germany; locality: Dresden; verbatimLocality: Dresden Botanical Garden; decimalLatitude: 51.0435; decimalLongitude: 13.7582; geodeticDatum: WGS84; **Event:** eventDate: 10 March 2013
- **Type status:** Other material. **Occurrence:** recordedBy: N. Lindner; individualCount: 2; disposition: SMNG; **Location:** country: Germany; locality: Leipzig; verbatimLocality: Leipzig Botanical Garden; decimalLatitude: 51.3283; decimalLongitude: 12.3912; geodeticDatum: WGS84; **Event:** eventDate: December 1995
- **Type status:** Other material. **Occurrence:** recordedBy: N. Lindner; individualCount: 1; disposition: SMNG; **Location:** country: Germany; locality: Leipzig; verbatimLocality: Zoological Garden Leipzig, Gondwanaland; decimalLatitude: 51.3505; decimalLongitude: 12.3716; geodeticDatum: WGS84; **Event:** eventDate: March - April 2013
- **Type status:** Other material. **Occurrence:** recordedBy: N. Lindner; individualCount: 1; disposition: SMNG; **Location:** country: Germany; locality: Potsdam; verbatimLocality: Biosphere Potsdam; decimalLatitude: 52.4185; decimalLongitude: 13.0486; geodeticDatum: WGS84; **Event:** eventDate: 19 August 2013

##### Distribution

Asia

#### Schendylidae

Cook, 1896

#### Pectiniunguis pauperatus

Silvestri, 1907

##### Materials

- **Type status:** Other material. **Occurrence:** individualCount: 1; **Location:** country: Germany; locality: Hamburg; verbatimLocality: Hamburg Botanical Garden; decimalLatitude: 53.5603; decimalLongitude: 9.9858; geodeticDatum: WGS84; **Record Level:** source: Silvestri 1907

##### Distribution

Asia

#### Diplopoda

de Blainville in Gervais, 1844

#### Julida

Brandt, 1833

#### Blaniulidae

C. L. Koch, 1847

#### Archiboreoiulus pallidus

(Brade-Birks, 1920)

##### Materials

- **Type status:** Other material. **Occurrence:** recordedBy: H. Reip; individualCount: 8; disposition: SMNG; **Location:** country: Germany; locality: Köln [Cologne]; verbatimLocality: Cologne Botanical Garden; decimalLatitude: 50.9607; decimalLongitude: 6.9692; geodeticDatum: WGS84; **Event:** eventDate: 30 June 2010

##### Distribution

Europe

#### Blaniulus guttulatus

(Bosc, 1792)

##### Materials

- **Type status:** Other material. **Occurrence:** recordedBy: Eichler; disposition: ZMB; **Location:** country: Germany; locality: Berlin; verbatimLocality: Berlin-Dahlem Botanical Garden; decimalLatitude: 52.4548; decimalLongitude: 13.3085; geodeticDatum: WGS84; **Record Level:** source: Eichler 1952
- **Type status:** Other material. **Occurrence:** recordedBy: N. Lindner; individualCount: 5; disposition: SMNG; **Location:** country: Germany; locality: Berlin; verbatimLocality: Berlin-Dahlem Botanical Garden; decimalLatitude: 52.4548; decimalLongitude: 13.3085; geodeticDatum: WGS84; **Event:** eventDate: 15 May 2013
- **Type status:** Other material. **Occurrence:** recordedBy: E. N. Lindner; individualCount: 5; disposition: SMNG; **Location:** country: Germany; locality: Berlin; verbatimLocality: Berlin-Dahlem Botanical Garden; decimalLatitude: 52.4548; decimalLongitude: 13.3085; geodeticDatum: WGS84; **Event:** eventDate: 15 May 2013
- **Type status:** Other material. **Occurrence:** recordedBy: P. Decker & S. Worch; individualCount: 1; disposition: SMNG; **Location:** country: Germany; locality: Dresden; verbatimLocality: Dresden Botanical Garden; decimalLatitude: 51.0435; decimalLongitude: 13.7582; geodeticDatum: WGS84; **Event:** eventDate: 07 January 2012
- **Type status:** Other material. **Occurrence:** recordedBy: N. Lindner; individualCount: 4; disposition: SMNG; **Location:** country: Germany; locality: Dresden; verbatimLocality: Dresden Botanical Garden; decimalLatitude: 51.0435; decimalLongitude: 13.7582; geodeticDatum: WGS84; **Event:** eventDate: 10 March 2013
- **Type status:** Other material. **Occurrence:** recordedBy: E. N. Lindner; individualCount: 4; disposition: SMNG; **Location:** country: Germany; locality: Dresden; verbatimLocality: Dresden Botanical Garden; decimalLatitude: 51.0435; decimalLongitude: 13.7582; geodeticDatum: WGS84; **Event:** eventDate: 10 March 2013
- **Type status:** Other material. **Occurrence:** recordedBy: E. Heußler; individualCount: 131; disposition: SMNG; **Location:** country: Germany; locality: Frankfurt am Main; verbatimLocality: Palm Garden; decimalLatitude: 50.1233; decimalLongitude: 8.6559; geodeticDatum: WGS84; **Event:** eventDate: 14-28 August 2008
- **Type status:** Other material. **Occurrence:** recordedBy: H. Reip; individualCount: 5; disposition: SMNG; **Location:** country: Germany; locality: Jena; verbatimLocality: Jena Botanical Garden; decimalLatitude: 50.9308; decimalLongitude: 11.5858; geodeticDatum: WGS84; **Event:** eventDate: 15 March 2004, 11 April 2008
- **Type status:** Other material. **Location:** country: Germany; locality: Köln [Cologne]; verbatimLocality: Zoological Institut at Weyertal; decimalLatitude: 50.9607; decimalLongitude: 6.9692; geodeticDatum: WGS84; **Record Level:** source: Thiele 1968
- **Type status:** Other material. **Occurrence:** recordedBy: H. Reip; individualCount: 2; disposition: SMNG; **Location:** country: Germany; locality: Potsdam; verbatimLocality: Potsdam Botanical Garden; decimalLatitude: 52.4040; decimalLongitude: 13.0250; geodeticDatum: WGS84; **Event:** eventDate: 07 November 2009
- **Type status:** Other material. **Occurrence:** individualCount: 1; **Location:** country: Germany; locality: Rostock; verbatimLocality: Rostock Botanical Garden; decimalLatitude: 54.0915; decimalLongitude: 12.0949; geodeticDatum: WGS84; **Event:** eventDate: 04 March 1926; **Record Level:** source: Schubart 1929b

##### Distribution

Europe

#### Boreoiulus tenuis

(Bigler, 1913)

##### Materials

- **Type status:** Other material. **Occurrence:** recordedBy: H. Reip; individualCount: 1; disposition: SMNG; **Location:** country: Germany; locality: Jena; verbatimLocality: Jena Botanical Garden; decimalLatitude: 50.9308; decimalLongitude: 11.5858; geodeticDatum: WGS84; **Event:** eventDate: 11 April 2008

##### Distribution

Europe

#### Choneiulus palmatus

(Nĕmec, 1895)

##### Materials

- **Type status:** Other material. **Occurrence:** disposition: ZMB; **Location:** country: Germany; locality: Berlin; verbatimLocality: Berlin-Dahlem Botanical Garden; decimalLatitude: 52.4548; decimalLongitude: 13.3085; geodeticDatum: WGS84; **Record Level:** source: Schubart 1929a, Eichler 1952
- **Type status:** Other material. **Occurrence:** recordedBy: H. Reip; individualCount: 12; disposition: SMNG; **Location:** country: Germany; locality: Berlin; verbatimLocality: Berlin-Dahlem Botanical Garden; decimalLatitude: 52.4548; decimalLongitude: 13.3085; geodeticDatum: WGS84; **Event:** eventDate: 19 March 2004, 29 April 2005
- **Type status:** Other material. **Occurrence:** recordedBy: N. Lindner; individualCount: 9; disposition: SMNG; **Location:** country: Germany; locality: Berlin; verbatimLocality: Berlin-Dahlem Botanical Garden; decimalLatitude: 52.4548; decimalLongitude: 13.3085; geodeticDatum: WGS84; **Event:** eventDate: 15 May 2013
- **Type status:** Other material. **Occurrence:** recordedBy: E. N. Lindner; individualCount: 9; disposition: SMNG; **Location:** country: Germany; locality: Berlin; verbatimLocality: Berlin-Dahlem Botanical Garden; decimalLatitude: 52.4548; decimalLongitude: 13.3085; geodeticDatum: WGS84; **Event:** eventDate: 15 May 2013
- **Type status:** Other material. **Occurrence:** recordedBy: C. Schmidt; **Location:** country: Germany; locality: Bochum; verbatimLocality: Bochum Botanical Garden; decimalLatitude: 51.4433; decimalLongitude: 7.2669; geodeticDatum: WGS84; **Event:** eventDate: November 2005
- **Type status:** Other material. **Location:** country: Germany; locality: Dresden; verbatimLocality: Dresden Botanical Garden; decimalLatitude: 51.0435; decimalLongitude: 13.7582; geodeticDatum: WGS84; **Event:** eventDate: 17 November 1964; **Record Level:** source: Richter 1967
- **Type status:** Other material. **Occurrence:** recordedBy: P. Decker & S. Worch; individualCount: 1; disposition: SMNG; **Location:** country: Germany; locality: Dresden; verbatimLocality: Dresden Botanical Garden; decimalLatitude: 51.0435; decimalLongitude: 13.7582; geodeticDatum: WGS84; **Event:** eventDate: 07 January 2012
- **Type status:** Other material. **Occurrence:** recordedBy: E. Heußler; individualCount: 19; disposition: SMNG; **Location:** country: Germany; locality: Frankfurt am Main; verbatimLocality: Palm Garden; decimalLatitude: 50.1233; decimalLongitude: 8.6559; geodeticDatum: WGS84; **Event:** eventDate: March-July 2008
- **Type status:** Other material. **Occurrence:** recordedBy: H. Reip; individualCount: 1; disposition: SMNG; **Location:** country: Germany; locality: Frankfurt am Main; verbatimLocality: Palm Garden; decimalLatitude: 50.1233; decimalLongitude: 8.6559; geodeticDatum: WGS84; **Event:** eventDate: 28 November 2011
- **Type status:** Other material. **Occurrence:** recordedBy: H. Reip; individualCount: 40; disposition: SMNG; **Location:** country: Germany; locality: Hamburg; verbatimLocality: Hamburg Botanical Garden; decimalLatitude: 53.5603; decimalLongitude: 9.9858; geodeticDatum: WGS84; **Event:** eventDate: 10 November 2007
- **Type status:** Other material. **Occurrence:** recordedBy: H. Reip; individualCount: 23; disposition: SMNG; **Location:** country: Germany; locality: Jena; verbatimLocality: Jena Botanical Garden; decimalLatitude: 50.9308; decimalLongitude: 11.5858; geodeticDatum: WGS84; **Event:** eventDate: 15 March 2004, 19 March 2004
- **Type status:** Other material. **Occurrence:** recordedBy: H. Reip; individualCount: 8; disposition: SMNG; **Location:** country: Germany; locality: Karlsruhe; verbatimLocality: Karlsruhe Botanical Garden; decimalLatitude: 49.0136; decimalLongitude: 8.4011; geodeticDatum: WGS84; **Event:** eventDate: 13 April 2010
- **Type status:** Other material. **Occurrence:** recordedBy: H. Reip; individualCount: 2; disposition: SMNG; **Location:** country: Germany; locality: Köln [Cologne]; verbatimLocality: Cologne Botanical Garden; decimalLatitude: 50.9607; decimalLongitude: 6.9692; geodeticDatum: WGS84; **Event:** eventDate: 30 June 2010
- **Type status:** Other material. **Location:** country: Germany; locality: Köln [Cologne]; verbatimLocality: Zoological Institut at Weyertal; decimalLatitude: 50.9607; decimalLongitude: 6.9692; geodeticDatum: WGS84; **Record Level:** source: Thiele 1968
- **Type status:** Other material. **Occurrence:** recordedBy: N. Lindner; individualCount: 2; disposition: SMNG; **Location:** country: Germany; locality: Leipzig; verbatimLocality: Leipzig Botanical Garden; decimalLatitude: 51.3283; decimalLongitude: 12.3912; geodeticDatum: WGS84; **Event:** eventDate: December 1999
- **Type status:** Other material. **Occurrence:** recordedBy: E. N. Lindner; individualCount: 2; disposition: SMNG; **Location:** country: Germany; locality: Leipzig; verbatimLocality: Leipzig Botanical Garden; decimalLatitude: 51.3283; decimalLongitude: 12.3912; geodeticDatum: WGS84; **Event:** eventDate: 12 December 1995
- **Type status:** Other material. **Occurrence:** recordedBy: H. Reip; individualCount: 33; disposition: SMNG; **Location:** country: Germany; locality: Magdeburg; verbatimLocality: Gruson-Greenhouses; decimalLatitude: 52.1139; decimalLongitude: 11.6315; geodeticDatum: WGS84; **Event:** eventDate: 13 June 2012
- **Type status:** Other material. **Occurrence:** recordedBy: P. Decker; individualCount: 1; disposition: SMNG; **Location:** country: Germany; locality: Mainz; verbatimLocality: Mainz Botanical Garden; decimalLatitude: 49.9925; decimalLongitude: 8.2454; geodeticDatum: WGS84; **Event:** eventDate: 04 November 2006
- **Type status:** Other material. **Occurrence:** recordedBy: H. Reip; individualCount: 25; disposition: SMNG; **Location:** country: Germany; locality: Potsdam; verbatimLocality: Potsdam Botanical Garden; decimalLatitude: 52.4040; decimalLongitude: 13.0250; geodeticDatum: WGS84; **Event:** eventDate: 07 November 2009
- **Type status:** Other material. **Occurrence:** individualCount: 1; **Location:** country: Germany; locality: Rostock; verbatimLocality: Rostock Botanical Garden; decimalLatitude: 54.0915; decimalLongitude: 12.0949; geodeticDatum: WGS84; **Event:** eventDate: 04 March 1926; **Record Level:** source: Schubart 1929b

##### Distribution

Europe

#### Nopoiulus kochii

(Gervais, 1847)

##### Materials

- **Type status:** Other material. **Occurrence:** recordedBy: P. Decker & M. Köhler; individualCount: 1; disposition: SMNG; **Location:** country: Germany; locality: Berlin; verbatimLocality: Berlin Zoological Garden; decimalLatitude: 52.5102; decimalLongitude: 13.3366; geodeticDatum: WGS84; **Event:** eventDate: 28 September 2013
- **Type status:** Other material. **Occurrence:** recordedBy: Eichler; disposition: ZMB; **Location:** country: Germany; locality: Berlin; verbatimLocality: Berlin-Dahlem Botanical Garden; decimalLatitude: 52.4548; decimalLongitude: 13.3085; geodeticDatum: WGS84; **Record Level:** source: Schubart 1929a, Eichler 1952
- **Type status:** Other material. **Occurrence:** recordedBy: N. Lindner; individualCount: 1; disposition: SMNG; **Location:** country: Germany; locality: Berlin; verbatimLocality: Berlin-Dahlem Botanical Garden; decimalLatitude: 52.4548; decimalLongitude: 13.3085; geodeticDatum: WGS84; **Event:** eventDate: 15 May 2013
- **Type status:** Other material. **Occurrence:** recordedBy: E. N. Lindner; individualCount: 1; disposition: SMNG; **Location:** country: Germany; locality: Berlin; verbatimLocality: Berlin-Dahlem Botanical Garden; decimalLatitude: 52.4548; decimalLongitude: 13.3085; geodeticDatum: WGS84; **Event:** eventDate: 15 May 2013
- **Type status:** Other material. **Occurrence:** recordedBy: E. Heußler; individualCount: 14; disposition: SMNG; **Location:** country: Germany; locality: Frankfurt am Main; verbatimLocality: Palm Garden; decimalLatitude: 50.1233; decimalLongitude: 8.6559; geodeticDatum: WGS84
- **Type status:** Other material. **Occurrence:** recordedBy: H. Reip; individualCount: 4; disposition: SMNG; **Location:** country: Germany; locality: Frankfurt am Main; verbatimLocality: Palm Garden; decimalLatitude: 50.1233; decimalLongitude: 8.6559; geodeticDatum: WGS84; **Event:** eventDate: 28 November 2011
- **Type status:** Other material. **Location:** country: Germany; locality: Kamen; verbatimLocality: hothouse near Kamen; decimalLatitude: 51.5900; decimalLongitude: 7.6600; geodeticDatum: WGS84; coordinateUncertaintyInMeters: 5800; **Event:** eventDate: 15 April 1905; **Record Level:** source: Verhoeff 1934
- **Type status:** Other material. **Occurrence:** recordedBy: Wilck, Adis & Golovatch; **Location:** country: Germany; locality: Kiel; verbatimLocality: Kiel Botanical Garden; decimalLatitude: 54.3470; decimalLongitude: 10.1158; geodeticDatum: WGS84; **Event:** eventDate: 07 November 1998–11 September 1999; **Record Level:** source: Wilck 2000
- **Type status:** Other material. **Occurrence:** recordedBy: H. Reip; individualCount: 26; disposition: SMNG; **Location:** country: Germany; locality: Potsdam; verbatimLocality: Potsdam Botanical Garden; decimalLatitude: 52.4040; decimalLongitude: 13.0250; geodeticDatum: WGS84; **Event:** eventDate: 07 November 2009
- **Type status:** Other material. **Occurrence:** disposition: ZMB; **Location:** country: Germany; locality: Potsdam; verbatimLocality: terrace nursery Park Sanssouci; decimalLatitude: 52.4040; decimalLongitude: 13.0250; geodeticDatum: WGS84; **Record Level:** source: Schubart 1929a

##### Distribution

Europe

#### Proteroiulus fuscus

(Am Stein, 1857)

##### Materials

- **Type status:** Other material. **Occurrence:** recordedBy: Boettger; **Location:** country: Germany; locality: Berlin; verbatimLocality: Berlin-Dahlem Botanical Garden; decimalLatitude: 52.4548; decimalLongitude: 13.3085; geodeticDatum: WGS84; **Record Level:** source: Boettger 1929
- **Type status:** Other material. **Occurrence:** recordedBy: H. Reip; individualCount: 3; disposition: SMNG; **Location:** country: Germany; locality: Potsdam; verbatimLocality: Potsdam Botanical Garden; decimalLatitude: 52.4040; decimalLongitude: 13.0250; geodeticDatum: WGS84; **Event:** eventDate: 07 November 2009

##### Distribution

Europe

#### Julidae

Leach, 1814

#### Brachyiulus pusillus

(Bosc, 1792)

##### Materials

- **Type status:** Other material. **Occurrence:** recordedBy: E. Heußler; individualCount: 1; disposition: SMNG; **Location:** country: Germany; locality: Frankfurt am Main; verbatimLocality: Palm Garden; decimalLatitude: 50.1233; decimalLongitude: 8.6559; geodeticDatum: WGS84; **Event:** eventDate: 28 March 2008-11 April 2008

##### Distribution

Europe

#### Cylindroiulus britannicus

(Verhoeff, 1891)

##### Materials

- **Type status:** Other material. **Occurrence:** recordedBy: Boettger; **Location:** country: Germany; locality: Berlin; verbatimLocality: Berlin-Dahlem Botanical Garden; decimalLatitude: 52.4548; decimalLongitude: 13.3085; geodeticDatum: WGS84; **Record Level:** source: Boettger 1929
- **Type status:** Other material. **Occurrence:** recordedBy: Eichler; disposition: ZMB; **Location:** country: Germany; locality: Berlin; verbatimLocality: Berlin-Dahlem Botanical Garden; decimalLatitude: 52.4548; decimalLongitude: 13.3085; geodeticDatum: WGS84; **Record Level:** source: Schubart 1929a, Eichler 1952
- **Type status:** Other material. **Occurrence:** recordedBy: N. Lindner; individualCount: 5; disposition: SMNG; **Location:** country: Germany; locality: Berlin; verbatimLocality: Berlin-Dahlem Botanical Garden; decimalLatitude: 52.4548; decimalLongitude: 13.3085; geodeticDatum: WGS84; **Event:** eventDate: 15 May 2013
- **Type status:** Other material. **Occurrence:** recordedBy: E. N. Lindner; individualCount: 5; disposition: SMNG; **Location:** country: Germany; locality: Berlin; verbatimLocality: Berlin-Dahlem Botanical Garden; decimalLatitude: 52.4548; decimalLongitude: 13.3085; geodeticDatum: WGS84; **Event:** eventDate: 15 May 2013
- **Type status:** Other material. **Occurrence:** recordedBy: Boettger; **Location:** country: Germany; locality: Berlin; verbatimLocality: horticulture company Bermann Rothe (Beyroth) in Berlin-Zehlendorf; decimalLatitude: 52.4320; decimalLongitude: 13.2580; geodeticDatum: WGS84; coordinateUncertaintyInMeters: 3000; **Record Level:** source: Boettger 1929
- **Type status:** Other material. **Occurrence:** recordedBy: P. Decker & N. Laufer; individualCount: 7; disposition: SMNG; **Location:** country: Germany; locality: Bonn; verbatimLocality: Bonn Botanical Garden; decimalLatitude: 50.7243; decimalLongitude: 7.0913; geodeticDatum: WGS84; **Event:** eventDate: 14 August 2007
- **Type status:** Other material. **Location:** country: Germany; locality: Dresden; verbatimLocality: Dresden Botanical Garden; decimalLatitude: 51.0435; decimalLongitude: 13.7582; geodeticDatum: WGS84; **Event:** eventDate: 17 November 1964; **Record Level:** source: Richter 1967
- **Type status:** Other material. **Occurrence:** recordedBy: P. Decker & S. Worch; disposition: SMNG; **Location:** country: Germany; locality: Dresden; verbatimLocality: Dresden Botanical Garden; decimalLatitude: 51.0435; decimalLongitude: 13.7582; geodeticDatum: WGS84; **Event:** eventDate: 07 January 2012
- **Type status:** Other material. **Occurrence:** recordedBy: H. Reip; individualCount: 6; disposition: SMNG; **Location:** country: Germany; locality: Hamburg; verbatimLocality: Hamburg Botanical Garden; decimalLatitude: 53.5603; decimalLongitude: 9.9858; geodeticDatum: WGS84; **Event:** eventDate: 10 November 2007
- **Type status:** Other material. **Occurrence:** individualCount: 45; **Location:** country: Germany; locality: Kamen; verbatimLocality: hothouse near Kamen; decimalLatitude: 51.5900; decimalLongitude: 7.6600; geodeticDatum: WGS84; coordinateUncertaintyInMeters: 5800; **Event:** eventDate: 15 April 1905; **Record Level:** source: Verhoeff 1934
- **Type status:** Other material. **Location:** country: Germany; locality: Lübeck; verbatimLocality: palm house; decimalLatitude: 53.8670; decimalLongitude: 10.6870; geodeticDatum: WGS84; coordinateUncertaintyInMeters: 5000; **Record Level:** source: Schubart 1925
- **Type status:** Other material. **Location:** country: Germany; locality: Potsdam; verbatimLocality: terrace nursery Park Sanssouci; decimalLatitude: 52.4040; decimalLongitude: 13.0250; geodeticDatum: WGS84; **Record Level:** source: Schubart 1929a

##### Distribution

Europe

#### Cylindroiulus caeruleocinctus

(Wood, 1864)

##### Materials

- **Type status:** Other material. **Occurrence:** recordedBy: H. Reip; individualCount: 1; disposition: SMNG; **Location:** country: Germany; locality: Berlin; verbatimLocality: Berlin-Dahlem Botanical Garden; decimalLatitude: 52.4548; decimalLongitude: 13.3085; geodeticDatum: WGS84; **Event:** eventDate: 29 April 2005
- **Type status:** Other material. **Occurrence:** recordedBy: Boettger; **Location:** country: Germany; locality: Berlin; verbatimLocality: Berlin-Dahlem Botanical Garden; decimalLatitude: 52.4548; decimalLongitude: 13.3085; geodeticDatum: WGS84; **Record Level:** source: Boettger 1929, Eichler 1952
- **Type status:** Other material. **Occurrence:** recordedBy: Boettger; **Location:** country: Germany; locality: Berlin; verbatimLocality: horticulture company Bermann Rothe (Beyroth) in Berlin-Zehlendorf; decimalLatitude: 52.4320; decimalLongitude: 13.2580; geodeticDatum: WGS84; coordinateUncertaintyInMeters: 3000; **Record Level:** source: Boettger 1929, Eichler 1952
- **Type status:** Other material. **Occurrence:** recordedBy: E. Heußler; individualCount: 11; disposition: SMNG; **Location:** country: Germany; locality: Frankfurt am Main; verbatimLocality: Palm Garden; decimalLatitude: 50.1233; decimalLongitude: 8.6559; geodeticDatum: WGS84; **Event:** eventDate: 11 April 2008-25 April 2008
- **Type status:** Other material. **Occurrence:** recordedBy: H. Reip; individualCount: 1; disposition: SMNG; **Location:** country: Germany; locality: Frankfurt am Main; verbatimLocality: Palm Garden; decimalLatitude: 50.1233; decimalLongitude: 8.6559; geodeticDatum: WGS84; **Event:** eventDate: 08 November 2008

##### Distribution

Europe

#### Cylindroiulus latestriatus

(Curtis, 1845)

##### Materials

- **Type status:** Other material. **Occurrence:** recordedBy: Eichler; individualCount: 2; disposition: ZMB; **Location:** country: Germany; locality: Berlin; verbatimLocality: Berlin-Dahlem Botanical Garden; decimalLatitude: 52.4548; decimalLongitude: 13.3085; geodeticDatum: WGS84; **Record Level:** source: Eichler 1952
- **Type status:** Other material. **Occurrence:** recordedBy: E. Heußler; individualCount: 3; disposition: SMNG; **Location:** country: Germany; locality: Frankfurt am Main; verbatimLocality: Palm Garden; decimalLatitude: 50.1233; decimalLongitude: 8.6559; geodeticDatum: WGS84; **Event:** eventDate: March 2008-May 2008
- **Type status:** Other material. **Occurrence:** individualCount: 2; **Location:** country: Germany; locality: Kiel; verbatimLocality: hothouse; decimalLatitude: 54.3200; decimalLongitude: 10.1400; geodeticDatum: WGS84; coordinateUncertaintyInMeters: 10000; **Event:** eventDate: 08 November 2008
- **Type status:** Other material. **Occurrence:** recordedBy: H. Reip; individualCount: 26; disposition: SMNG; **Location:** country: Germany; locality: Potsdam; verbatimLocality: Potsdam Botanical Garden; decimalLatitude: 52.4040; decimalLongitude: 13.0250; geodeticDatum: WGS84; **Event:** eventDate: 07 November 2009
- **Type status:** Other material. **Occurrence:** recordedBy: H. Reip; individualCount: 6; disposition: SMNG; **Location:** country: Germany; locality: Oldenburg; verbatimLocality: Oldenburg Botanical Garden; decimalLatitude: 53.1486; decimalLongitude: 8.1942; geodeticDatum: WGS84; **Event:** eventDate: 29 November 2013

##### Distribution

Europe

#### Cylindroiulus punctatus

(Leach, 1815)

##### Materials

- **Type status:** Other material. **Occurrence:** recordedBy: P. Decker & M. Köhler; individualCount: 1; disposition: SMNG; **Location:** country: Germany; locality: Berlin; verbatimLocality: Berlin Zoological Garden; decimalLatitude: 52.5102; decimalLongitude: 13.3366; geodeticDatum: WGS84; **Event:** eventDate: 28 September 2013
- **Type status:** Other material. **Location:** country: Germany; locality: Berlin; verbatimLocality: Berlin Old Botanical Garden; decimalLatitude: 52.4565; decimalLongitude: 13.3074; geodeticDatum: WGS84; **Record Level:** source: Eichler 1952

##### Distribution

Europe

#### Cylindroiulus truncorum

(Silvestri, 1896)

##### Materials

- **Type status:** Other material. **Occurrence:** recordedBy: P. Decker & M. Köhler; individualCount: 1; disposition: SMNG; **Location:** country: Germany; locality: Berlin; verbatimLocality: Berlin Zoological Garden; decimalLatitude: 52.5102; decimalLongitude: 13.3366; geodeticDatum: WGS84; **Event:** eventDate: 28 September 2013
- **Type status:** Other material. **Occurrence:** recordedBy: H. Reip; individualCount: 39; disposition: SMNG; **Location:** country: Germany; locality: Berlin; verbatimLocality: Berlin-Dahlem Botanical Garden; decimalLatitude: 52.4548; decimalLongitude: 13.3085; geodeticDatum: WGS84; **Event:** eventDate: 19 March 2004, 29 April 2005
- **Type status:** Other material. **Occurrence:** individualCount: >3; disposition: ZMB; **Location:** country: Germany; locality: Berlin; verbatimLocality: Berlin-Dahlem Botanical Garden; decimalLatitude: 52.4548; decimalLongitude: 13.3085; geodeticDatum: WGS84; **Record Level:** source: Schubart 1929a, Boettger 1929, Eichler 1952
- **Type status:** Other material. **Occurrence:** recordedBy: E. N. Lindner; individualCount: 10; disposition: SMNG; **Location:** country: Germany; locality: Berlin; verbatimLocality: Berlin-Dahlem Botanical Garden; decimalLatitude: 52.4548; decimalLongitude: 13.3085; geodeticDatum: WGS84; **Event:** eventDate: 15 May 2013
- **Type status:** Other material. **Occurrence:** recordedBy: P. Decker & S. Worch; individualCount: 8; disposition: SMNG; **Location:** country: Germany; locality: Dresden; verbatimLocality: Dresden Botanical Garden; decimalLatitude: 51.0435; decimalLongitude: 13.7582; geodeticDatum: WGS84; **Event:** eventDate: 07 January 2012
- **Type status:** Other material. **Occurrence:** recordedBy: E. N. Lindner; individualCount: 2; disposition: SMNG; **Location:** country: Germany; locality: Dresden; verbatimLocality: Dresden Botanical Garden; decimalLatitude: 51.0435; decimalLongitude: 13.7582; geodeticDatum: WGS84; **Event:** eventDate: 10 March 2013
- **Type status:** Other material. **Occurrence:** recordedBy: E. Heußler; individualCount: 17; disposition: SMNG; **Location:** country: Germany; locality: Frankfurt am Main; verbatimLocality: Palm Garden; decimalLatitude: 50.1233; decimalLongitude: 8.6559; geodeticDatum: WGS84; **Event:** eventDate: 14 March 2008-17 July 2008
- **Type status:** Other material. **Occurrence:** recordedBy: H. Reip; individualCount: 23; disposition: SMNG; **Location:** country: Germany; locality: Frankfurt am Main; verbatimLocality: Palm Garden; decimalLatitude: 50.1233; decimalLongitude: 8.6559; geodeticDatum: WGS84; **Event:** eventDate: 08 November 2008, 28 November 2011
- **Type status:** Other material. **Occurrence:** recordedBy: H. Reip; individualCount: 11; disposition: SMNG; **Location:** country: Germany; locality: Jena; verbatimLocality: Jena Botanical Garden; decimalLatitude: 50.9308; decimalLongitude: 11.5858; geodeticDatum: WGS84; **Event:** eventDate: 11 April 2008
- **Type status:** Other material. **Occurrence:** individualCount: 4; **Location:** country: Germany; locality: Kamen; verbatimLocality: hothouse near Kamen; decimalLatitude: 51.5900; decimalLongitude: 7.6600; geodeticDatum: WGS84; coordinateUncertaintyInMeters: 5800; **Event:** eventDate: 15 April 1905; **Record Level:** source: Verhoeff 1934
- **Type status:** Other material. **Occurrence:** recordedBy: H. Reip; individualCount: 57; disposition: SMNG; **Location:** country: Germany; locality: Karlsruhe; verbatimLocality: Karlsruhe Botanical Garden; decimalLatitude: 49.0136; decimalLongitude: 8.4011; geodeticDatum: WGS84; **Event:** eventDate: 13 April 2010
- **Type status:** Other material. **Occurrence:** recordedBy: Wilck, Adis & Golovatch; **Location:** country: Germany; locality: Kiel; verbatimLocality: Kiel Botanical Garden; decimalLatitude: 54.3470; decimalLongitude: 10.1158; geodeticDatum: WGS84; **Event:** eventDate: 07 November 1998–11 September 1999
- **Type status:** Other material. **Occurrence:** recordedBy: P. Decker & N. Laufer; individualCount: 3; disposition: SMNG; **Location:** country: Germany; locality: Köln [Cologne]; verbatimLocality: Cologne Botanical Garden; decimalLatitude: 50.9607; decimalLongitude: 6.9692; geodeticDatum: WGS84; **Event:** eventDate: 14 August 2007
- **Type status:** Other material. **Occurrence:** recordedBy: H. Reip; individualCount: 13; disposition: SMNG; **Location:** country: Germany; locality: Köln [Cologne]; verbatimLocality: Cologne Botanical Garden; decimalLatitude: 50.9607; decimalLongitude: 6.9692; geodeticDatum: WGS84; **Event:** eventDate: 30 June 2010
- **Type status:** Other material. **Occurrence:** recordedBy: N. Lindner; individualCount: 13; disposition: SMNG; **Location:** country: Germany; locality: Leipzig; verbatimLocality: nursery garden and horticulture Felgenträger; decimalLatitude: 51.3690; decimalLongitude: 12.4400; geodeticDatum: WGS84; **Event:** eventDate: 09 November 2008
- **Type status:** Other material. **Occurrence:** recordedBy: E. N. Lindner; individualCount: 1; disposition: SMNG; **Location:** country: Germany; locality: Leipzig; verbatimLocality: Zoological Garden Leipzig, Gondwanaland; decimalLatitude: 51.3505; decimalLongitude: 12.3716; geodeticDatum: WGS84; **Event:** eventDate: 26 March 2013
- **Type status:** Other material. **Occurrence:** recordedBy: H. Reip; individualCount: 30; disposition: SMNG; **Location:** country: Germany; locality: Magdeburg; verbatimLocality: Gruson-Greenhouses; decimalLatitude: 52.1139; decimalLongitude: 11.6315; geodeticDatum: WGS84; **Event:** eventDate: 13 June 2012
- **Type status:** Other material. **Occurrence:** recordedBy: P. Decker; individualCount: 3; disposition: SMNG; **Location:** country: Germany; locality: Mainz; verbatimLocality: Mainz Botanical Garden; decimalLatitude: 49.9925; decimalLongitude: 8.2454; geodeticDatum: WGS84; **Event:** eventDate: 04 November 2006
- **Type status:** Other material. **Occurrence:** recordedBy: H. Reip; disposition: SMNG; **Location:** country: Germany; locality: München [Munich]; verbatimLocality: München-Nymphenburg Botanical Garden; decimalLatitude: 48.1606; decimalLongitude: 11.5021; geodeticDatum: WGS84; **Event:** eventDate: 28 May 2009
- **Type status:** Other material. **Occurrence:** recordedBy: H. Reip; individualCount: 40; disposition: SMNG; **Location:** country: Germany; locality: Potsdam; verbatimLocality: Potsdam Botanical Garden; decimalLatitude: 52.4040; decimalLongitude: 13.0250; geodeticDatum: WGS84; **Event:** eventDate: 07 November 2009

##### Distribution

North Africa

#### Cylindroiulus vulnerarius

(Berlese, 1888)

##### Materials

- **Type status:** Other material. **Occurrence:** recordedBy: H. Reip; individualCount: 1; disposition: SMNG; **Location:** country: Germany; locality: Potsdam; verbatimLocality: Potsdam Botanical Garden; decimalLatitude: 52.4040; decimalLongitude: 13.0250; geodeticDatum: WGS84; **Event:** eventDate: 07 November 2009
- **Type status:** Other material. **Occurrence:** recordedBy: H. Reip; individualCount: 2; disposition: SMNG; **Location:** country: Germany; locality: Potsdam; verbatimLocality: Potsdam Botanical Garden; decimalLatitude: 52.4040; decimalLongitude: 13.0250; geodeticDatum: WGS84; **Event:** eventDate: 07 November 2009

##### Distribution

Europe

#### Kryphioiulus occultus

(C. L. Koch, 1847)

##### Materials

- **Type status:** Other material. **Occurrence:** recordedBy: Eichler; individualCount: 2; disposition: ZMB; **Location:** country: Germany; locality: Berlin; verbatimLocality: Berlin-Dahlem Botanical Garden; decimalLatitude: 52.4548; decimalLongitude: 13.3085; geodeticDatum: WGS84; **Record Level:** source: Eichler 1952
- **Type status:** Other material. **Occurrence:** recordedBy: E. N. Lindner; individualCount: 1; disposition: SMNG; **Location:** country: Germany; locality: Berlin; verbatimLocality: Berlin-Dahlem Botanical Garden; decimalLatitude: 52.4548; decimalLongitude: 13.3085; geodeticDatum: WGS84; **Event:** eventDate: 15 May 2013

##### Distribution

Europe

#### Mesoiulus gridellii

Strasser, 1934

##### Materials

- **Type status:** Other material. **Occurrence:** recordedBy: E. Heußler; individualCount: 1; disposition: SMNG; **Location:** country: Germany; locality: Frankfurt am Main; verbatimLocality: Palm Garden; decimalLatitude: 50.1233; decimalLongitude: 8.6559; geodeticDatum: WGS84; **Event:** eventDate: 11 April 2008-25 April 2008

##### Distribution

Europe

#### Ommatoiulus sabulosus

(Linnaeus, 1758)

##### Materials

- **Type status:** Other material. **Location:** country: Germany; locality: Berlin; verbatimLocality: Berlin-Dahlem Botanical Garden; decimalLatitude: 52.4548; decimalLongitude: 13.3085; geodeticDatum: WGS84; **Record Level:** source: Eichler 1952

##### Distribution

Europe

#### Unciger foetidus

(C. L. Koch, 1838)

##### Materials

- **Type status:** Other material. **Location:** country: Germany; locality: Berlin; verbatimLocality: Berlin-Dahlem Botanical Garden; decimalLatitude: 52.4548; decimalLongitude: 13.3085; geodeticDatum: WGS84; **Record Level:** source: Eichler 1952

##### Distribution

Europe

#### Spirobolida

Cook, 1895

#### Rhinocricidae

Brölemann, 1913

#### Anadenobolus monilicornis

(Porat, 1876)

##### Materials

- **Type status:** Other material. **Occurrence:** recordedBy: E. N. Lindner; individualCount: 6; disposition: SMNG; **Location:** country: Germany; locality: Leipzig; verbatimLocality: Zoological Garden Leipzig, Gondwanaland; decimalLatitude: 51.3505; decimalLongitude: 12.3716; geodeticDatum: WGS84; **Event:** eventDate: 26 March 2013, 05 April 2013, 28 July 2013

##### Distribution

Central America, South America

#### Spirobolellidae

Bollman, 1893

#### Paraspirobolus lucifugus

(Gervais, 1836)

##### Materials

- **Type status:** Other material. **Occurrence:** individualCount: 1; disposition: ZMB; **Location:** country: Germany; locality: Berlin; verbatimLocality: Berlin-Dahlem Botanical Garden; decimalLatitude: 52.4548; decimalLongitude: 13.3085; geodeticDatum: WGS84; **Record Level:** source: Schubart 1929a, Schubart 1934
- **Type status:** Other material. **Occurrence:** recordedBy: Eichler; individualCount: 6; disposition: ZMB; **Location:** country: Germany; locality: Berlin; verbatimLocality: Berlin-Dahlem Botanical Garden; decimalLatitude: 52.4548; decimalLongitude: 13.3085; geodeticDatum: WGS84; **Record Level:** source: Eichler 1952
- **Type status:** Other material. **Occurrence:** recordedBy: E. N. Lindner; individualCount: 21; disposition: SMNG; **Location:** country: Germany; locality: Berlin; verbatimLocality: Berlin-Dahlem Botanical Garden; decimalLatitude: 52.4548; decimalLongitude: 13.3085; geodeticDatum: WGS84; **Event:** eventDate: 15 May 2013
- **Type status:** Other material. **Occurrence:** recordedBy: P. Decker & N. Laufer; individualCount: 5; disposition: SMNG; **Location:** country: Germany; locality: Bonn; verbatimLocality: Bonn Botanical Garden; decimalLatitude: 50.7243; decimalLongitude: 7.0913; geodeticDatum: WGS84; **Event:** eventDate: 14 August 2007; **Record Level:** source: Decker & Hannig 2011
- **Type status:** Other material. **Occurrence:** recordedBy: H. Reip; individualCount: 1; disposition: SMNG; **Location:** country: Germany; locality: Frankfurt am Main; verbatimLocality: Palm Garden; decimalLatitude: 50.1233; decimalLongitude: 8.6559; geodeticDatum: WGS84; **Event:** eventDate: 28 November 2011
- **Type status:** Other material. **Occurrence:** recordedBy: H. Reip; disposition: SMNG; **Location:** country: Germany; locality: Hamburg; verbatimLocality: Hamburg Botanical Garden; decimalLatitude: 53.5603; decimalLongitude: 9.9858; geodeticDatum: WGS84; **Event:** eventDate: 10 November 2007
- **Type status:** Other material. **Location:** country: Germany; locality: Hamburg; verbatimLocality: tannery; decimalLatitude: 53.4870; decimalLongitude: 10.2150; geodeticDatum: WGS84; coordinateUncertaintyInMeters: 4000; **Record Level:** source: Latzel 1895
- **Type status:** Other material. **Occurrence:** recordedBy: E. N. Lindner; individualCount: 3; disposition: SMNG; **Location:** country: Germany; locality: Leipzig; verbatimLocality: Zoological Garden Leipzig, Gondwanaland; decimalLatitude: 51.3505; decimalLongitude: 12.3716; geodeticDatum: WGS84; **Event:** eventDate: 26 March 2013, 05 April 2013

##### Distribution

South America

#### Trigoniulidae

Attems, 1909

#### Leptogoniulus sorornus

(Butler, 1876)

##### Materials

- **Type status:** Other material. **Occurrence:** recordedBy: P. Decker & M. Köhler; individualCount: 1; disposition: SMNG; **Location:** country: Germany; locality: Berlin; verbatimLocality: Berlin Zoological Garden; decimalLatitude: 52.5102; decimalLongitude: 13.3366; geodeticDatum: WGS84; **Event:** eventDate: 28 September 2013
- **Type status:** Other material. **Occurrence:** recordedBy: E. N. Lindner; individualCount: 33; disposition: SMNG; **Location:** country: Germany; locality: Leipzig; verbatimLocality: Zoological Garden Leipzig, Gondwanaland; decimalLatitude: 51.3505; decimalLongitude: 12.3716; geodeticDatum: WGS84; **Event:** eventDate: 26 March 2013, 05 April 2013, 28 July 2013

##### Distribution

Southeast Asia

#### Spirostreptida

Brandt, 1833

#### Epinannolenidae

Silvestri, 1895

#### Epinannolene cf. trinidadensis

Chamberlin, 1918

##### Materials

- **Type status:** Other material. **Occurrence:** recordedBy: E. N. Lindner; individualCount: 16; disposition: SMNG; **Location:** country: Germany; locality: Leipzig; verbatimLocality: Zoological Garden Leipzig, Gondwanaland; decimalLatitude: 51.3505; decimalLongitude: 12.3716; geodeticDatum: WGS84; **Event:** eventDate: 26 March 2013, 05 April 2013

##### Distribution

South America

#### Epinannolene sp.

##### Materials

- **Type status:** Other material. **Occurrence:** recordedBy: E. Heußler; individualCount: 12; disposition: SMNG; **Location:** country: Germany; locality: Frankfurt am Main; verbatimLocality: Palm Garden; decimalLatitude: 50.1233; decimalLongitude: 8.6559; geodeticDatum: WGS84; **Event:** eventDate: 28 March 2008-24 October 2008
- **Type status:** Other material. **Occurrence:** recordedBy: H. Reip; individualCount: 23; disposition: SMNG; **Location:** country: Germany; locality: Frankfurt am Main; verbatimLocality: Palm Garden; decimalLatitude: 50.1233; decimalLongitude: 8.6559; geodeticDatum: WGS84; **Event:** eventDate: 08 November 2008, 28 November 2011

##### Distribution

South America

#### Polyzoniida

Cook, 1895

#### Siphonotidae

Cook, 1895

#### Rhinotus purpureus

(Pocock, 1894)

##### Materials

- **Type status:** Other material. **Occurrence:** recordedBy: E. Heußler; individualCount: 35; disposition: SMNG; **Location:** country: Germany; locality: Frankfurt am Main; verbatimLocality: Palm Garden; decimalLatitude: 50.1233; decimalLongitude: 8.6559; geodeticDatum: WGS84; **Event:** eventDate: 14 March 2008-20 August 2008
- **Type status:** Other material. **Occurrence:** recordedBy: H. Reip; individualCount: 1; disposition: SMNG; **Location:** country: Germany; locality: Frankfurt am Main; verbatimLocality: Palm Garden; decimalLatitude: 50.1233; decimalLongitude: 8.6559; geodeticDatum: WGS84; **Event:** eventDate: 28 November 2011

##### Distribution

South America

#### Polydesmida

Pocock, 1887

#### Haplodesmidae

Cook, 1895

#### Cylindrodesmus hirsutus

Pocock, 1889

##### Materials

- **Type status:** Other material. **Occurrence:** recordedBy: P. Decker & M. Köhler; disposition: SMNG; **Location:** country: Germany; locality: Berlin; verbatimLocality: Berlin Zoological Garden; decimalLatitude: 52.5102; decimalLongitude: 13.3366; geodeticDatum: WGS84; **Event:** eventDate: 28 September 2013
- **Type status:** Other material. **Occurrence:** recordedBy: H. Reip; individualCount: 4; disposition: SMNG; **Location:** country: Germany; locality: Berlin; verbatimLocality: Berlin-Dahlem Botanical Garden; decimalLatitude: 52.4548; decimalLongitude: 13.3085; geodeticDatum: WGS84; **Event:** eventDate: 29 April 2005
- **Type status:** Other material. **Occurrence:** recordedBy: M. Zerm; **Location:** country: Germany; locality: Berlin; verbatimLocality: Berlin-Dahlem Botanical Garden; decimalLatitude: 52.4548; decimalLongitude: 13.3085; geodeticDatum: WGS84; **Event:** eventDate: May 2000, August 2000
- **Type status:** Other material. **Occurrence:** recordedBy: E. N. Lindner; individualCount: 34; disposition: SMNG; **Location:** country: Germany; locality: Berlin; verbatimLocality: Berlin-Dahlem Botanical Garden; decimalLatitude: 52.4548; decimalLongitude: 13.3085; geodeticDatum: WGS84; **Event:** eventDate: 15 May 2013
- **Type status:** Other material. **Occurrence:** recordedBy: E. Heußler; individualCount: 2; disposition: SMNG; **Location:** country: Germany; locality: Frankfurt am Main; verbatimLocality: Palm Garden; decimalLatitude: 50.1233; decimalLongitude: 8.6559; geodeticDatum: WGS84
- **Type status:** Other material. **Occurrence:** recordedBy: H. Reip; individualCount: 16; disposition: SMNG; **Location:** country: Germany; locality: Frankfurt am Main; verbatimLocality: Palm Garden; decimalLatitude: 50.1233; decimalLongitude: 8.6559; geodeticDatum: WGS84; **Event:** eventDate: 28 November 2011
- **Type status:** Other material. **Occurrence:** recordedBy: H. Reip; individualCount: 6; disposition: SMNG; **Location:** country: Germany; locality: Hamburg; verbatimLocality: Hamburg Botanical Garden; decimalLatitude: 53.5603; decimalLongitude: 9.9858; geodeticDatum: WGS84; **Event:** eventDate: 10 November 2007
- **Type status:** Other material. **Occurrence:** recordedBy: P. Decker & N. Laufer; individualCount: 2; disposition: SMNG; **Location:** country: Germany; locality: Köln [Cologne]; verbatimLocality: Cologne Botanical Garden; decimalLatitude: 50.9607; decimalLongitude: 6.9692; geodeticDatum: WGS84; **Event:** eventDate: 14 August 2007
- **Type status:** Other material. **Occurrence:** recordedBy: H. Reip; individualCount: 1; disposition: SMNG; **Location:** country: Germany; locality: Köln [Cologne]; verbatimLocality: Cologne Botanical Garden; decimalLatitude: 50.9607; decimalLongitude: 6.9692; geodeticDatum: WGS84; **Event:** eventDate: 30 June 2010
- **Type status:** Other material. **Occurrence:** recordedBy: E. N. Lindner; individualCount: 11; disposition: SMNG; **Location:** country: Germany; locality: Leipzig; verbatimLocality: Leipzig Botanical Garden; decimalLatitude: 51.3283; decimalLongitude: 12.3912; geodeticDatum: WGS84; **Event:** eventDate: 03 April 2013
- **Type status:** Other material. **Occurrence:** recordedBy: E. N. Lindner; individualCount: 31; disposition: SMNG; **Location:** country: Germany; locality: Leipzig; verbatimLocality: Zoological Garden Leipzig, Gondwanaland; decimalLatitude: 51.3505; decimalLongitude: 12.3716; geodeticDatum: WGS84; **Event:** eventDate: 26 March 2013, 05 April 2013
- **Type status:** Other material. **Occurrence:** recordedBy: P. Decker; individualCount: 10; disposition: SMNG; **Location:** country: Germany; locality: Mainz; verbatimLocality: Mainz Botanical Garden; decimalLatitude: 49.9925; decimalLongitude: 8.2454; geodeticDatum: WGS84; **Event:** eventDate: 04 November 2006
- **Type status:** Other material. **Occurrence:** recordedBy: E. N. Lindner; individualCount: 5; disposition: SMNG; **Location:** country: Germany; locality: Potsdam; verbatimLocality: Biosphere Potsdam; decimalLatitude: 52.4185; decimalLongitude: 13.0486; geodeticDatum: WGS84; **Event:** eventDate: 19 September 2013
- **Type status:** Other material. **Occurrence:** recordedBy: H. Reip; individualCount: 24; disposition: SMNG; **Location:** country: Germany; locality: Potsdam; verbatimLocality: Potsdam Botanical Garden; decimalLatitude: 52.4040; decimalLongitude: 13.0250; geodeticDatum: WGS84; **Event:** eventDate: 07 November 2009
- **Type status:** Other material. **Occurrence:** recordedBy: H. Reip; individualCount: 4; disposition: SMNG; **Location:** country: Germany; locality: Oldenburg; verbatimLocality: Oldenburg Botanical Garden; decimalLatitude: 53.1486; decimalLongitude: 8.1942; geodeticDatum: WGS84; **Event:** eventDate: 29 November 2013

##### Distribution

Asia

#### Prosopodesmus jacobsoni

Silvestri, 1910

##### Materials

- **Type status:** Other material. **Occurrence:** recordedBy: P. Decker & N. Laufer; individualCount: 8; disposition: SMNG; **Location:** country: Germany; locality: Bonn; verbatimLocality: Bonn Botanical Garden; decimalLatitude: 50.7243; decimalLongitude: 7.0913; geodeticDatum: WGS84; **Event:** eventDate: 14 August 2007; **Record Level:** source: Decker & Hannig 2011
- **Type status:** Other material. **Occurrence:** recordedBy: E. Heußler; individualCount: 51; disposition: SMNG; **Location:** country: Germany; locality: Frankfurt am Main; verbatimLocality: Palm Garden; decimalLatitude: 50.1233; decimalLongitude: 8.6559; geodeticDatum: WGS84; **Event:** eventDate: 14 March 2008-24 October 2008; **Record Level:** source: Decker & Hannig 2011
- **Type status:** Other material. **Occurrence:** recordedBy: H. Reip; individualCount: 2; disposition: SMNG; **Location:** country: Germany; locality: Frankfurt am Main; verbatimLocality: Palm Garden; decimalLatitude: 50.1233; decimalLongitude: 8.6559; geodeticDatum: WGS84; **Event:** eventDate: 28 November 2011; **Record Level:** source: Decker & Hannig 2011
- **Type status:** Other material. **Occurrence:** recordedBy: P. Decker & N. Laufer; individualCount: 2; disposition: SMNG; **Location:** country: Germany; locality: Köln [Cologne]; verbatimLocality: Cologne Botanical Garden; decimalLatitude: 50.9607; decimalLongitude: 6.9692; geodeticDatum: WGS84; **Event:** eventDate: 14 August 2007; **Record Level:** source: Decker & Hannig 2011
- **Type status:** Other material. **Occurrence:** recordedBy: H. Reip; individualCount: 11; disposition: SMNG; **Location:** country: Germany; locality: Köln [Cologne]; verbatimLocality: Cologne Botanical Garden; decimalLatitude: 50.9607; decimalLongitude: 6.9692; geodeticDatum: WGS84; **Event:** eventDate: 30 June 2010; **Record Level:** source: Decker & Hannig 2011

##### Distribution

Indo-Australian region?

#### Oniscodesmidae

DeSaussure, 1860

#### Amphitomeus attemsi

(Schubart, 1934)

##### Materials

- **Type status:** Other material. **Occurrence:** recordedBy: H. Reip; individualCount: 5; disposition: SMNG; **Location:** country: Germany; locality: Berlin; verbatimLocality: Berlin-Dahlem Botanical Garden; decimalLatitude: 52.4548; decimalLongitude: 13.3085; geodeticDatum: WGS84; **Event:** eventDate: 29 April 2005
- **Type status:** Other material. **Occurrence:** recordedBy: Eichler; disposition: ZMB; **Location:** country: Germany; locality: Berlin; verbatimLocality: Berlin-Dahlem Botanical Garden; decimalLatitude: 52.4548; decimalLongitude: 13.3085; geodeticDatum: WGS84; **Record Level:** source: Eichler 1952, Schubart 1934
- **Type status:** Other material. **Occurrence:** recordedBy: E. N. Lindner; individualCount: 12; disposition: SMNG; **Location:** country: Germany; locality: Berlin; verbatimLocality: Berlin-Dahlem Botanical Garden; decimalLatitude: 52.4548; decimalLongitude: 13.3085; geodeticDatum: WGS84; **Event:** eventDate: 15 May 2013
- **Type status:** Other material. **Occurrence:** individualCount: 4; **Location:** country: Germany; locality: Bochum; verbatimLocality: Bochum Botanical Garden; decimalLatitude: 51.4433; decimalLongitude: 7.2669; geodeticDatum: WGS84; **Event:** eventDate: 25 June 1905
- **Type status:** Other material. **Occurrence:** recordedBy: P. Decker & N. Laufer; individualCount: 1; disposition: SMNG; **Location:** country: Germany; locality: Bonn; verbatimLocality: Bonn Botanical Garden; decimalLatitude: 50.7243; decimalLongitude: 7.0913; geodeticDatum: WGS84; **Event:** eventDate: 14 August 2007; **Record Level:** source: Decker & Hannig 2011
- **Type status:** Other material. **Occurrence:** recordedBy: E. Heußler; individualCount: 2; disposition: SMNG; **Location:** country: Germany; locality: Frankfurt am Main; verbatimLocality: Palm Garden; decimalLatitude: 50.1233; decimalLongitude: 8.6559; geodeticDatum: WGS84; **Event:** eventDate: 03 July 2008-17 July 2008
- **Type status:** Other material. **Occurrence:** recordedBy: H. Reip; individualCount: 15; disposition: SMNG; **Location:** country: Germany; locality: Halle (Saale); verbatimLocality: Halle Botanical Garden; decimalLatitude: 51.4894; decimalLongitude: 11.9591; geodeticDatum: WGS84; **Event:** eventDate: 09 May 2011
- **Type status:** Other material. **Occurrence:** recordedBy: H. Reip; individualCount: 11; disposition: SMNG; **Location:** country: Germany; locality: Hamburg; verbatimLocality: Hamburg Botanical Garden; decimalLatitude: 53.5603; decimalLongitude: 9.9858; geodeticDatum: WGS84; **Event:** eventDate: 10 November 2007
- **Type status:** Other material. **Occurrence:** recordedBy: H. Reip; individualCount: 6; disposition: SMNG; **Location:** country: Germany; locality: Jena; verbatimLocality: Jena Botanical Garden; decimalLatitude: 50.9308; decimalLongitude: 11.5858; geodeticDatum: WGS84; **Event:** eventDate: 15 March 2004, 11 April 2008
- **Type status:** Other material. **Occurrence:** recordedBy: H. Reip; individualCount: 11; disposition: SMNG; **Location:** country: Germany; locality: Karlsruhe; verbatimLocality: Karlsruhe Botanical Garden; decimalLatitude: 49.0136; decimalLongitude: 8.4011; geodeticDatum: WGS84; **Event:** eventDate: 13 April 2010
- **Type status:** Other material. **Occurrence:** recordedBy: Wilck, Adis & Golovatch; **Location:** country: Germany; locality: Kiel; verbatimLocality: Kiel Botanical Garden; decimalLatitude: 54.3470; decimalLongitude: 10.1158; geodeticDatum: WGS84; **Event:** eventDate: 07 November 1998–11 September 1999; **Record Level:** source: Wilck 2000
- **Type status:** Other material. **Occurrence:** recordedBy: P. Decker & N. Laufer; individualCount: 4; disposition: SMNG; **Location:** country: Germany; locality: Köln [Cologne]; verbatimLocality: Cologne Botanical Garden; decimalLatitude: 50.9607; decimalLongitude: 6.9692; geodeticDatum: WGS84; **Event:** eventDate: 14 August 2007; **Record Level:** source: Decker & Hannig 2011
- **Type status:** Other material. **Occurrence:** recordedBy: H. Reip; individualCount: 10; disposition: SMNG; **Location:** country: Germany; locality: Köln [Cologne]; verbatimLocality: Cologne Botanical Garden; decimalLatitude: 50.9607; decimalLongitude: 6.9692; geodeticDatum: WGS84; **Event:** eventDate: 30 June 2010
- **Type status:** Other material. **Occurrence:** recordedBy: E. N. Lindner; individualCount: 23; disposition: SMNG; **Location:** country: Germany; locality: Leipzig; verbatimLocality: Leipzig Botanical Garden; decimalLatitude: 51.3283; decimalLongitude: 12.3912; geodeticDatum: WGS84; **Event:** eventDate: 31 December 1995, 03 April 2013
- **Type status:** Other material. **Occurrence:** recordedBy: H. Reip; individualCount: 29; disposition: SMNG; **Location:** country: Germany; locality: Magdeburg; verbatimLocality: Gruson-Greenhouses; decimalLatitude: 52.1139; decimalLongitude: 11.6315; geodeticDatum: WGS84; **Event:** eventDate: 13 June 2012
- **Type status:** Other material. **Occurrence:** recordedBy: P. Decker; individualCount: 8; disposition: SMNG; **Location:** country: Germany; locality: Mainz; verbatimLocality: Mainz Botanical Garden; decimalLatitude: 49.9925; decimalLongitude: 8.2454; geodeticDatum: WGS84; **Event:** eventDate: 04 November 2006
- **Type status:** Other material. **Occurrence:** recordedBy: H. Reip; individualCount: 6; disposition: SMNG; **Location:** country: Germany; locality: München [Munich]; verbatimLocality: München-Nymphenburg Botanical Garden; decimalLatitude: 48.1606; decimalLongitude: 11.5021; geodeticDatum: WGS84; **Event:** eventDate: 28 May 2009
- **Type status:** Other material. **Occurrence:** recordedBy: E. N. Lindner; individualCount: 5; disposition: SMNG; **Location:** country: Germany; locality: Potsdam; verbatimLocality: Biosphere Potsdam; decimalLatitude: 52.4185; decimalLongitude: 13.0486; geodeticDatum: WGS84; **Event:** eventDate: 19 September 2013
- **Type status:** Other material. **Occurrence:** recordedBy: H. Reip; individualCount: 33; disposition: SMNG; **Location:** country: Germany; locality: Potsdam; verbatimLocality: Potsdam Botanical Garden; decimalLatitude: 52.4040; decimalLongitude: 13.0250; geodeticDatum: WGS84; **Event:** eventDate: 07 November 2009

##### Distribution

South America

#### Paradoxosomatidae

Daday, 1889

#### Oxidus gracilis

(C. L. Koch, 1847)

##### Materials

- **Type status:** Other material. **Occurrence:** recordedBy: A. Berthold; individualCount: 1; **Location:** country: Germany; locality: Bayreuth; verbatimLocality: Bayreuth Ecological Botanical Garden; decimalLatitude: 50.9810; decimalLongitude: 13.5730; geodeticDatum: WGS84; coordinateUncertaintyInMeters: 500; **Event:** eventDate: 23 January 2012
- **Type status:** Other material. **Occurrence:** recordedBy: H. Hennig; individualCount: 1; disposition: SMNG; **Location:** country: Germany; locality: Bayreuth; verbatimLocality: Bayreuth Ecological Botanical Garden; decimalLatitude: 50.9810; decimalLongitude: 13.5730; geodeticDatum: WGS84; coordinateUncertaintyInMeters: 500; **Event:** eventDate: 16 February 2012
- **Type status:** Other material. **Occurrence:** recordedBy: P. Decker & M. Köhler; individualCount: 7; disposition: SMNG; **Location:** country: Germany; locality: Berlin; verbatimLocality: Berlin Zoological Garden; decimalLatitude: 52.5102; decimalLongitude: 13.3366; geodeticDatum: WGS84; **Event:** eventDate: 28 September 2013
- **Type status:** Other material. **Occurrence:** recordedBy: H. Reip; individualCount: 5; disposition: SMNG; **Location:** country: Germany; locality: Berlin; verbatimLocality: Berlin-Dahlem Botanical Garden; decimalLatitude: 52.4548; decimalLongitude: 13.3085; geodeticDatum: WGS84; **Event:** eventDate: 29 April 2005
- **Type status:** Other material. **Occurrence:** recordedBy: Boettger; **Location:** country: Germany; locality: Berlin; verbatimLocality: Berlin-Dahlem Botanical Garden; decimalLatitude: 52.4548; decimalLongitude: 13.3085; geodeticDatum: WGS84; **Event:** eventDate: 1927-1928; **Record Level:** source: Boettger 1929
- **Type status:** Other material. **Occurrence:** disposition: ZMB; **Location:** country: Germany; locality: Berlin; verbatimLocality: Berlin-Dahlem Botanical Garden; decimalLatitude: 52.4548; decimalLongitude: 13.3085; geodeticDatum: WGS84; **Record Level:** source: Schubart 1929a, Schubart 1934
- **Type status:** Other material. **Occurrence:** recordedBy: Dahl; **Location:** country: Germany; locality: Berlin; verbatimLocality: Berlin-Dahlem Botanical Garden; decimalLatitude: 52.4548; decimalLongitude: 13.3085; geodeticDatum: WGS84; **Record Level:** source: Verhoeff 1907
- **Type status:** Other material. **Occurrence:** recordedBy: Eichler; **Location:** country: Germany; locality: Berlin; verbatimLocality: Berlin-Dahlem Botanical Garden; decimalLatitude: 52.4548; decimalLongitude: 13.3085; geodeticDatum: WGS84; **Record Level:** source: Eichler 1952
- **Type status:** Other material. **Occurrence:** recordedBy: E. N. Lindner; individualCount: 28; disposition: SMNG; **Location:** country: Germany; locality: Berlin; verbatimLocality: Berlin-Dahlem Botanical Garden; decimalLatitude: 52.4548; decimalLongitude: 13.3085; geodeticDatum: WGS84; **Event:** eventDate: 15 May 2013
- **Type status:** Other material. **Location:** country: Germany; locality: Berlin; verbatimLocality: cemetary nursery in Eythstraße; decimalLatitude: 52.4590; decimalLongitude: 13.3640; geodeticDatum: WGS84; coordinateUncertaintyInMeters: 250; **Record Level:** source: Schubart 1957
- **Type status:** Other material. **Occurrence:** recordedBy: Boettger; **Location:** country: Germany; locality: Berlin; verbatimLocality: horticulture company Bermann Rothe (Beyroth) in Berlin-Zehlendorf; decimalLatitude: 52.4320; decimalLongitude: 13.2580; geodeticDatum: WGS84; coordinateUncertaintyInMeters: 3000; **Record Level:** source: Boettger 1929
- **Type status:** Other material. **Location:** country: Germany; locality: Berlin; verbatimLocality: nursery; decimalLatitude: 52.5200; decimalLongitude: 13.3900; geodeticDatum: WGS84; coordinateUncertaintyInMeters: 20000; **Record Level:** source: Schubart 1929a
- **Type status:** Other material. **Occurrence:** recordedBy: C. Schmidt; **Location:** country: Germany; locality: Bochum; verbatimLocality: Bochum Botanical Garden; decimalLatitude: 51.4433; decimalLongitude: 7.2669; geodeticDatum: WGS84; **Event:** eventDate: November 2005
- **Type status:** Other material. **Occurrence:** recordedBy: P. Decker & N. Laufer; individualCount: 5; disposition: SMNG; **Location:** country: Germany; locality: Bonn; verbatimLocality: Bonn Botanical Garden; decimalLatitude: 50.7243; decimalLongitude: 7.0913; geodeticDatum: WGS84; **Event:** eventDate: 14 August 2007
- **Type status:** Other material. **Occurrence:** recordedBy: K. W. Verhoeff; disposition: ZMB; **Location:** country: Germany; locality: Bonn; verbatimLocality: hothouse of Mr. Biesing; decimalLatitude: 50.7315; decimalLongitude: 7.0977; geodeticDatum: WGS84; coordinateUncertaintyInMeters: 3240; **Event:** eventDate: 08 August 1890; **Record Level:** source: Verhoeff 1891
- **Type status:** Other material. **Occurrence:** recordedBy: P. Decker & S. Worch; individualCount: 12; disposition: SMNG; **Location:** country: Germany; locality: Dresden; verbatimLocality: Dresden Botanical Garden; decimalLatitude: 51.0435; decimalLongitude: 13.7582; geodeticDatum: WGS84; **Event:** eventDate: 07 January 2012
- **Type status:** Other material. **Occurrence:** recordedBy: H. Richter; individualCount: 35; disposition: SMNG; **Location:** country: Germany; locality: Dresden; verbatimLocality: Dresden Botanical Garden; decimalLatitude: 51.0435; decimalLongitude: 13.7582; geodeticDatum: WGS84; **Event:** eventDate: 13 November 1964, 17 November 1964, 21 November 1964; **Record Level:** source: Richter 1967
- **Type status:** Other material. **Occurrence:** recordedBy: E. N. Lindner; individualCount: 8; disposition: SMNG; **Location:** country: Germany; locality: Dresden; verbatimLocality: Dresden Botanical Garden; decimalLatitude: 51.0435; decimalLongitude: 13.7582; geodeticDatum: WGS84; **Event:** eventDate: 10 March 2013
- **Type status:** Other material. **Occurrence:** recordedBy: Beckers; individualCount: 1; **Location:** country: Germany; locality: Duisburg; verbatimLocality: greenhouse; decimalLatitude: 51.4300; decimalLongitude: 6.7600; geodeticDatum: WGS84; coordinateUncertaintyInMeters: 10000; **Event:** eventDate: 20 November 1987
- **Type status:** Other material. **Occurrence:** recordedBy: E. Heußler; individualCount: 34; disposition: SMNG; **Location:** country: Germany; locality: Frankfurt am Main; verbatimLocality: Palm Garden; decimalLatitude: 50.1233; decimalLongitude: 8.6559; geodeticDatum: WGS84; **Event:** eventDate: 06 June 2008-17 July 2008
- **Type status:** Other material. **Occurrence:** recordedBy: H. Reip; individualCount: 8; disposition: SMNG; **Location:** country: Germany; locality: Frankfurt am Main; verbatimLocality: Palm Garden; decimalLatitude: 50.1233; decimalLongitude: 8.6559; geodeticDatum: WGS84; **Event:** eventDate: 08 November 2008, 28 November 2011
- **Type status:** Other material. **Occurrence:** individualCount: 9; **Location:** country: Germany; locality: Gießen; verbatimLocality: Gießen Botanical Garden; decimalLatitude: 50.5864; decimalLongitude: 8.6789; geodeticDatum: WGS84; **Event:** eventDate: 1973-1974; **Record Level:** source: Läffert 1984
- **Type status:** Other material. **Occurrence:** recordedBy: H. Reip; individualCount: 14; disposition: SMNG; **Location:** country: Germany; locality: Halle (Saale); verbatimLocality: Halle Botanical Garden; decimalLatitude: 51.4894; decimalLongitude: 11.9591; geodeticDatum: WGS84; **Event:** eventDate: 09 May 2011
- **Type status:** Other material. **Location:** country: Germany; locality: Halle (Saale; verbatimLocality: hothouse; decimalLatitude: 51.4790; decimalLongitude: 11.9640; geodeticDatum: WGS84; coordinateUncertaintyInMeters: 6000; **Record Level:** source: Schubart 1934
- **Type status:** Other material. **Occurrence:** individualCount: 1; **Location:** country: Germany; locality: Hamburg; verbatimLocality: hothouses of the nursery Richers; decimalLatitude: 53.5500; decimalLongitude: 9.9900; geodeticDatum: WGS84; coordinateUncertaintyInMeters: 10000; **Record Level:** source: Latzel 1895
- **Type status:** Other material. **Occurrence:** recordedBy: H. Reip; individualCount: 1; disposition: SMNG; **Location:** country: Germany; locality: Hamburg; verbatimLocality: Hamburg Botanical Garden; decimalLatitude: 53.5603; decimalLongitude: 9.9858; geodeticDatum: WGS84; **Event:** eventDate: 10 November 2007
- **Type status:** Other material. **Location:** country: Germany; locality: Hannover; verbatimLocality: hothouse; decimalLatitude: 52.3700; decimalLongitude: 9.7400; geodeticDatum: WGS84; coordinateUncertaintyInMeters: 10000; **Record Level:** source: Schubart 1934
- **Type status:** Other material. **Occurrence:** recordedBy: H. Reip; individualCount: 15; disposition: SMNG; **Location:** country: Germany; locality: Jena; verbatimLocality: Jena Botanical Garden; decimalLatitude: 50.9308; decimalLongitude: 11.5858; geodeticDatum: WGS84; **Event:** eventDate: 15 March 2004, 19 March 2004, 11 April 2008
- **Type status:** Other material. **Occurrence:** recordedBy: H. Reip; individualCount: 12; disposition: SMNG; **Location:** country: Germany; locality: Karlsruhe; verbatimLocality: Karlsruhe Botanical Garden; decimalLatitude: 49.0136; decimalLongitude: 8.4011; geodeticDatum: WGS84; **Event:** eventDate: 13 April 2010
- **Type status:** Other material. **Occurrence:** recordedBy: Wilck, Adis & Golovatch; **Location:** country: Germany; locality: Kiel; verbatimLocality: Kiel Botanical Garden; decimalLatitude: 54.3470; decimalLongitude: 10.1158; geodeticDatum: WGS84; **Event:** eventDate: 07 November 1998–11 September 1999; **Record Level:** source: Wilck 2000
- **Type status:** Other material. **Occurrence:** recordedBy: H. Reip; individualCount: 8; disposition: SMNG; **Location:** country: Germany; locality: Köln [Cologne]; verbatimLocality: Cologne Botanical Garden; decimalLatitude: 50.9607; decimalLongitude: 6.9692; geodeticDatum: WGS84; **Event:** eventDate: 30 June 2010
- **Type status:** Other material. **Occurrence:** recordedBy: N. Lindner; individualCount: 1; disposition: SMNG; **Location:** country: Germany; locality: Konstanz; verbatimLocality: Mainau, butterfly house; decimalLatitude: 47.7068; decimalLongitude: 9.1951; geodeticDatum: WGS84; **Event:** eventDate: 21 April 2012
- **Type status:** Other material. **Occurrence:** recordedBy: E. N. Lindner; disposition: SMNG; **Location:** country: Germany; locality: Leipzig; verbatimLocality: Leipzig Botanical Garden; decimalLatitude: 51.3283; decimalLongitude: 12.3912; geodeticDatum: WGS84; **Event:** eventDate: 30 November 1995
- **Type status:** Other material. **Occurrence:** recordedBy: N. Lindner; individualCount: 6; disposition: SMNG; **Location:** country: Germany; locality: Leipzig; verbatimLocality: nursery garden and horticulture Felgenträger; decimalLatitude: 51.3690; decimalLongitude: 12.4400; geodeticDatum: WGS84; **Event:** eventDate: 09 November 2008
- **Type status:** Other material. **Occurrence:** recordedBy: E. N. Lindner; individualCount: 5; disposition: SMNG; **Location:** country: Germany; locality: Leipzig; verbatimLocality: Zoological Garden Leipzig, Gondwanaland; decimalLatitude: 51.3505; decimalLongitude: 12.3716; geodeticDatum: WGS84; **Event:** eventDate: 26 March 2013, 05 April 2013
- **Type status:** Other material. **Occurrence:** recordedBy: H. Reip; individualCount: 23; disposition: SMNG; **Location:** country: Germany; locality: Magdeburg; verbatimLocality: Gruson-Greenhouses; decimalLatitude: 52.1139; decimalLongitude: 11.6315; geodeticDatum: WGS84; **Event:** eventDate: 13 June 2012
- **Type status:** Other material. **Occurrence:** recordedBy: P. Decker; individualCount: 28; disposition: SMNG; **Location:** country: Germany; locality: Mainz; verbatimLocality: Mainz Botanical Garden; decimalLatitude: 49.9925; decimalLongitude: 8.2454; geodeticDatum: WGS84; **Event:** eventDate: 04 November 2006
- **Type status:** Other material. **Occurrence:** recordedBy: P. Decker; disposition: SMNG; **Location:** country: Germany; locality: Mainz; verbatimLocality: Palm house at city park; decimalLatitude: 49.9897; decimalLongitude: 8.2893; geodeticDatum: WGS84; **Event:** eventDate: 1998-2009
- **Type status:** Other material. **Occurrence:** recordedBy: M. Reich & S. Schaub-Grüssing; individualCount: 34; disposition: SMNG; **Location:** country: Germany; locality: Marburg; verbatimLocality: Marburg Botanical Garden; decimalLatitude: 50.8024; decimalLongitude: 8.8078; geodeticDatum: WGS84; **Event:** eventDate: 15 February 2012
- **Type status:** Other material. **Occurrence:** recordedBy: H. Reip; individualCount: 3; disposition: SMNG; **Location:** country: Germany; locality: Marlow; verbatimLocality: Ornithological Park Marlow; decimalLatitude: 54.1449; decimalLongitude: 12.5692; geodeticDatum: WGS84; **Event:** eventDate: 31 July 2006
- **Type status:** Other material. **Location:** country: Germany; locality: München [Munich]; verbatimLocality: München-Nymphenburg Botanical Garden; decimalLatitude: 48.1606; decimalLongitude: 11.5021; geodeticDatum: WGS84; **Record Level:** source: Boettger 1929
- **Type status:** Other material. **Occurrence:** recordedBy: H. Hauser; disposition: SMNG; **Location:** country: Germany; locality: Potsdam; verbatimLocality: Biosphere Potsdam; decimalLatitude: 52.4185; decimalLongitude: 13.0486; geodeticDatum: WGS84; **Event:** eventDate: 20 June 1905
- **Type status:** Other material. **Occurrence:** recordedBy: H. Reip; individualCount: 25; disposition: SMNG; **Location:** country: Germany; locality: Potsdam; verbatimLocality: Potsdam Botanical Garden; decimalLatitude: 52.4040; decimalLongitude: 13.0250; geodeticDatum: WGS84; **Event:** eventDate: 07 November 2009
- **Type status:** Other material. **Location:** country: Germany; locality: Potsdam; verbatimLocality: horticulture company Bermann Rothe (Beyroth) in Potsdam-Neubabelsberg; decimalLatitude: 52.3990; decimalLongitude: 13.1040; geodeticDatum: WGS84; coordinateUncertaintyInMeters: 3000; **Record Level:** source: Schubart 1957
- **Type status:** Other material. **Occurrence:** recordedBy: H. Richter; individualCount: 2; disposition: SMNG; **Location:** country: Germany; locality: Tharandt; verbatimLocality: Tharandt Forest Botanical Garden; decimalLatitude: 50.9810; decimalLongitude: 13.5730; geodeticDatum: WGS84; coordinateUncertaintyInMeters: 500; **Event:** eventDate: 23 July 1966
- **Type status:** Other material. **Occurrence:** recordedBy: D. Matzke; individualCount: 1; **Location:** country: Germany; locality: Leipzig; verbatimLocality: Leipzig Botanical Garden; decimalLatitude: 51.3283; decimalLongitude: 12.3912; geodeticDatum: WGS84; **Event:** eventDate: 18 March 2013
- **Type status:** Other material. **Occurrence:** recordedBy: H. Reip; individualCount: 11; disposition: SMNG; **Location:** country: Germany; locality: Oldenburg; verbatimLocality: Oldenburg Botanical Garden; decimalLatitude: 53.1486; decimalLongitude: 8.1942; geodeticDatum: WGS84; **Event:** eventDate: 29 November 2013

##### Distribution

East Asia

#### Strongylosoma stigmatosum

(Eichwald, 1830)

##### Materials

- **Type status:** Other material. **Location:** country: Germany; locality: Berlin; verbatimLocality: Berlin-Dahlem Botanical Garden; decimalLatitude: 52.4548; decimalLongitude: 13.3085; geodeticDatum: WGS84; **Record Level:** source: Eichler 1952

##### Distribution

Europe

#### Polydesmidae

Leach, 1815

#### Brachydesmus superus

Latzel, 1884

##### Materials

- **Type status:** Other material. **Occurrence:** recordedBy: H. Reip; individualCount: 1; disposition: SMNG; **Location:** country: Germany; locality: Berlin; verbatimLocality: Berlin-Dahlem Botanical Garden; decimalLatitude: 52.4548; decimalLongitude: 13.3085; geodeticDatum: WGS84; **Event:** eventDate: 19 March 2004
- **Type status:** Other material. **Occurrence:** recordedBy: Eichler; disposition: ZMB; **Location:** country: Germany; locality: Berlin; verbatimLocality: Berlin-Dahlem Botanical Garden; decimalLatitude: 52.4548; decimalLongitude: 13.3085; geodeticDatum: WGS84; **Record Level:** source: Schubart 1929a, Eichler 1952
- **Type status:** Other material. **Location:** country: Germany; locality: Berlin; verbatimLocality: Berlin-Dahlem Botanical Garden; decimalLatitude: 52.4548; decimalLongitude: 13.3085; geodeticDatum: WGS84; **Record Level:** source: Eichler 1952

##### Distribution

Europe

#### Polydesmus angustus

Latzel, 1884

##### Materials

- **Type status:** Other material. **Occurrence:** recordedBy: P. Decker & S. Worch; individualCount: 1; disposition: SMNG; **Location:** country: Germany; locality: Dresden; verbatimLocality: Dresden Botanical Garden; decimalLatitude: 51.0435; decimalLongitude: 13.7582; geodeticDatum: WGS84; **Event:** eventDate: 07 January 2012

##### Distribution

Europe

#### Polydesmus complanatus

(Linnaeus, 1761)

##### Materials

- **Type status:** Other material. **Location:** country: Germany; locality: Hamburg; verbatimLocality: Hamburg-Wandsbeck; decimalLatitude: 53.5830; decimalLongitude: 10.0850; geodeticDatum: WGS84; coordinateUncertaintyInMeters: 1000; **Record Level:** source: Hahmann 1929a,Hahmann 1929b
- **Type status:** Other material. **Location:** country: Germany; locality: Berlin; verbatimLocality: Berlin Old Botanical Garden; decimalLatitude: 52.4565; decimalLongitude: 13.3074; geodeticDatum: WGS84; **Record Level:** source: Eichler 1952

##### Distribution

Europe

#### Polydesmus inconstans

Latzel, 1884

##### Materials

- **Type status:** Other material. **Location:** country: Germany; locality: Berlin; verbatimLocality: Berlin-Dahlem Botanical Garden; decimalLatitude: 52.4548; decimalLongitude: 13.3085; geodeticDatum: WGS84; **Record Level:** source: Eichler 1952
- **Type status:** Other material. **Occurrence:** recordedBy: E. N. Lindner; individualCount: 5; disposition: SMNG; **Location:** country: Germany; locality: Berlin; verbatimLocality: Berlin-Dahlem Botanical Garden; decimalLatitude: 52.4548; decimalLongitude: 13.3085; geodeticDatum: WGS84; **Event:** eventDate: 15 May 2013

##### Distribution

Europe

#### Pyrgodesmidae

Silvestri, 1896

#### Poratia digitata

(Porat, 1889)

##### Materials

- **Type status:** Other material. **Occurrence:** recordedBy: H. Reip; individualCount: 2; disposition: SMNG; **Location:** country: Germany; locality: Berlin; verbatimLocality: Berlin-Dahlem Botanical Garden; decimalLatitude: 52.4548; decimalLongitude: 13.3085; geodeticDatum: WGS84; **Event:** eventDate: 29 April 2005
- **Type status:** Other material. **Location:** country: Germany; locality: Berlin; verbatimLocality: Berlin-Dahlem Botanical Garden; decimalLatitude: 52.4548; decimalLongitude: 13.3085; geodeticDatum: WGS84; **Record Level:** source: Eichler 1952
- **Type status:** Other material. **Occurrence:** individualCount: 1; disposition: ZMB; **Location:** country: Germany; locality: Berlin; verbatimLocality: Berlin-Dahlem Botanical Garden; decimalLatitude: 52.4548; decimalLongitude: 13.3085; geodeticDatum: WGS84; **Record Level:** source: Schubart 1929a
- **Type status:** Other material. **Location:** country: Germany; locality: Dresden; verbatimLocality: Dresden Botanical Garden; decimalLatitude: 51.0435; decimalLongitude: 13.7582; geodeticDatum: WGS84; **Event:** eventDate: 17 November 1964; **Record Level:** source: Richter 1967
- **Type status:** Other material. **Location:** country: Germany; locality: Düsseldorf; verbatimLocality: Aquazoo-Löbbecke Museum; decimalLatitude: 51.2564; decimalLongitude: 6.7497; geodeticDatum: WGS84; **Record Level:** source: Adis et al. 2000
- **Type status:** Other material. **Occurrence:** recordedBy: H. Reip; individualCount: 7; disposition: SMNG; **Location:** country: Germany; locality: Hamburg; verbatimLocality: Hamburg Botanical Garden; decimalLatitude: 53.5603; decimalLongitude: 9.9858; geodeticDatum: WGS84; **Event:** eventDate: 10 November 2007
- **Type status:** Other material. **Occurrence:** individualCount: 3; **Location:** country: Germany; locality: Hamburg; verbatimLocality: tannery; decimalLatitude: 53.4870; decimalLongitude: 10.2150; geodeticDatum: WGS84; coordinateUncertaintyInMeters: 4000; **Record Level:** source: Latzel 1895
- **Type status:** Other material. **Occurrence:** recordedBy: H. Reip; individualCount: 7; disposition: SMNG; **Location:** country: Germany; locality: Karlsruhe; verbatimLocality: Karlsruhe Botanical Garden; decimalLatitude: 49.0136; decimalLongitude: 8.4011; geodeticDatum: WGS84; **Event:** eventDate: 13 April 2010
- **Type status:** Other material. **Occurrence:** recordedBy: Wilck, Adis & Golovatch; individualCount: 2; **Location:** country: Germany; locality: Kiel; verbatimLocality: Kiel Botanical Garden; decimalLatitude: 54.3470; decimalLongitude: 10.1158; geodeticDatum: WGS84; **Event:** eventDate: 07 November 1998–11 September 1999; **Record Level:** source: Wilck 2000, Adis et al. 2000
- **Type status:** Other material. **Occurrence:** recordedBy: E. N. Lindner; individualCount: 12; disposition: SMNG; **Location:** country: Germany; locality: Leipzig; verbatimLocality: Leipzig Botanical Garden; decimalLatitude: 51.3283; decimalLongitude: 12.3912; geodeticDatum: WGS84; **Event:** eventDate: 08 December 1995, 31 December 1995
- **Type status:** Other material. **Occurrence:** recordedBy: E. N. Lindner; individualCount: 6; disposition: SMNG; **Location:** country: Germany; locality: Leipzig; verbatimLocality: Zoological Garden Leipzig, Gondwanaland; decimalLatitude: 51.3505; decimalLongitude: 12.3716; geodeticDatum: WGS84; **Event:** eventDate: 26 March 2013, 05 April 2013
- **Type status:** Other material. **Occurrence:** recordedBy: H. Reip; individualCount: 53; disposition: SMNG; **Location:** country: Germany; locality: Potsdam; verbatimLocality: Potsdam Botanical Garden; decimalLatitude: 52.4040; decimalLongitude: 13.0250; geodeticDatum: WGS84; **Event:** eventDate: 07 November 2009
- **Type status:** Other material. **Occurrence:** recordedBy: H. Reip; individualCount: 27; disposition: SMNG; **Location:** country: Germany; locality: Oldenburg; verbatimLocality: Oldenburg Botanical Garden; decimalLatitude: 53.1486; decimalLongitude: 8.1942; geodeticDatum: WGS84; **Event:** eventDate: 29 November 2013

##### Distribution

South America

#### Poratia obliterata

(Kraus, 1960)

##### Materials

- **Type status:** Other material. **Occurrence:** recordedBy: Wilck, Adis & Golovatch; individualCount: 20; **Location:** country: Germany; locality: Kiel; verbatimLocality: Kiel Botanical Garden; decimalLatitude: 54.3470; decimalLongitude: 10.1158; geodeticDatum: WGS84; **Event:** eventDate: 07 November 1998–11 September 1999; **Record Level:** source: Wilck 2000, Adis et al. 2000

##### Distribution

South America

#### Chordeumatida

Pocock 1894

#### Chordeumatidae

C. L. Koch, 1847

#### Melogona voigtii

(Verhoeff, 1899)

##### Materials

- **Type status:** Other material. **Location:** country: Germany; locality: Berlin; verbatimLocality: Berlin-Dahlem Botanical Garden; decimalLatitude: 52.4548; decimalLongitude: 13.3085; geodeticDatum: WGS84; **Record Level:** source: Eichler 1952

##### Distribution

Europe

## Discussion

The present study is the second comprehensive survey of German greenhouse myriapods since Eichler (1952) and is derived from both the existing literature and our own extensive sampling.Eichler (1952) We collected more than 1800 specimens belonging to 41 species (29 Diplopoda, 12 Chilopoda), which makes a total of 53 species (35 Diplopoda and 18 Chilopoda) (Suppl. material 2).

**Figure 3.**
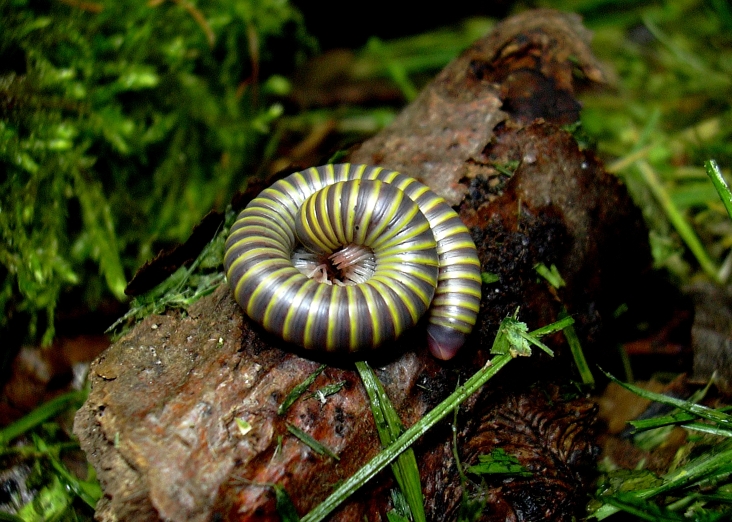
The colourful Caribbean species *Anadenobolus monilicornis* was only recorded in Gondwanaland, Zoological Garden Leipzig.

Six centipedes and 12 millipedes are herewith recorded for the first time from German greenhouses: *Cryptops doriae*, *Henia vesuviana*, *Lamyctes coeculus*, *Lithobius mutabilis*, *Pachymerium ferrugineum*, *Tygarrup javanicus*, *Anadenobolus monilicornis* (Fig. 3), *Archiboreoiulus pallidus*, *Boreoiulus tenuis*, *Brachyiulus pusillus*, *Cylindroiulus vulnerarius*, Epinannolene cf. trinidadensis, *Epinannolene* spec., *Leptogonoiulus sorornus*, *Mesoiulus gridellii*, *Polydesmus angustus*, *Prosopodesmus jacobsoni* and *Rhinotus purpureus*.

**Figure 4.**
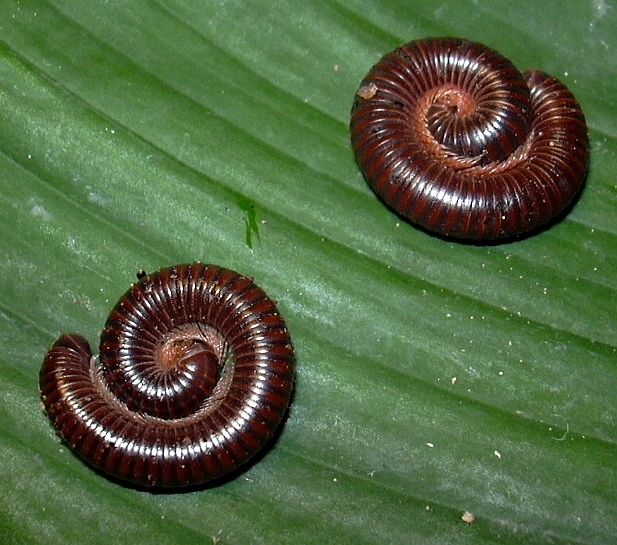
The tropical tramp species *Leptogoniulus sorornus* is here recorded for the first time in Europe from the Zoological Garden Leipzig and the Zoological Garden Berlin.

*Leptogonoiulus sorornus* (Fig. 4) and the two species of genus *Epinannolene* are recorded for the first time in Europe. While the first species of *Epinannolene* fits quite well the original description of *Epinannolene trinidanensis* by Chamberlin (1918), the second one is probably an undescribed species, closely related to *Epinannolene alticola* (Silvestri, 1898) and *Epinannolene exilio* (Brölemann, 1904).

A total of 34% (i.e. 18 species) are species introduced from other continents (Suppl. material 2): 15 % of those recorded have their origin in Southern and Central America, 13% in Asia, 4 % in Australia, and only 2 % in Africa.

The total number of non-European species introduced to Germany is higher and includes, for instance, Chondrodesmus cf. riparius Carl, 1914 in houseplants (Decker and Hannig 2011) and other occasionally introduced species (Attems 1901, Dunger 1965, Jeekel 1964b, Kraepelin 1901, Schubart 1934 pp. 304-306). Stoev et al. (2010) list for Europe 20 alien species of millipedes and 16 of centipedes.

Most of the alien species in German greenhouses are also known from other such places in Europe. There are a few species recorded from other European greenhouses which we have not found in Germany yet. These could also be expected to possibly be introduced to or occur in German greenhouses – species such as *Mecistocephalus guildingii* (Barber 2009), *Prosopodesmus panporus* Blower & Rundle, 1980 (Blower and Rundle 1980), and *Haplopodoiulus spathifer* (Brölemann, 1897) (Read 2008) recorded in Great Britain, *Tuoba poseidonis* (Verhoeff, 1901) recorded in Finland (Andersson et al. 2005) and *Aulonopygus aculeatus* Attems, 1914 recorded from the Netherlands (Soesbergen and Jeekel 2007).

Sixty-six per cent of all recorded species (35 in total) are of European origin. Two examples of the successful establishment of indigenous species in greenhouses are *Lithobius forficatus* and *Blaniulus guttulatus*. *Lithobius forficatus*, followed by *Cryptops hortensis*, is the most frequently found centipede in German greenhouses. The former normally inhabits natural habitats and areas of human settlement and is frequently found in houses and cellars. Likewise, among indigenous millipedes *Blaniulus guttulatus* is the most commonly found. In Germany it prefers synanthropic habitats and occurs on arable and waste land, while, especially in the west and southwest of Germany, it is also known from human settlements (Schubart 1934).

About 25% of the recorded species are found exclusively in greenhouses, especially in heated ones, and are thus unlikely to establish outside. These are: *Cryptops doriae*, *Lamyctes coeculus*, *Tygarrup javanicus*, *Amphitomeus attemsi*, *Anadenobolus monilicornis*, *Cylindrodesmus hirsutus*, *Epinannolene* species, *Leptogoniulus sorornus*, *Mesoiulus gridellii*, *Paraspirobolus lucifugus*, *Poratia* species, *Prosopodesmus jacobsoni*, and *Rhinotus purpureus*.

**Figure 5.**
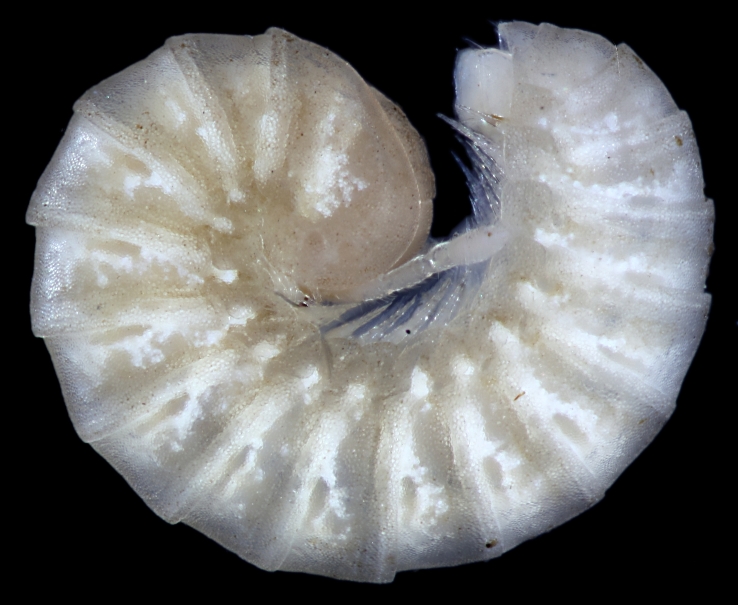
The tiny (2.9-3.2 mm) parthenogenetic *Amphitomeus attemsi* is a common species in greenhouses.

The parthenogenetic species *Amphitomeus attemsi* (Fig. 5), *Cylindrodesmus hirsutus* and *Poratia digitata* are very frequent (with *Amphitomeus attemsi* being the second most common millipede species) and were found in many of the greenhouses investigated.

The East Asian species *Oxidus gracilis*, which is the most frequent millipede in greenhouses, was observed outdoors in two sites in Mainz, Rhineland-Palatinate (2003-2009, pers. obs. Decker) during winter, albeit only in very large compost heaps or large accumulations of rotting material with evenly warm-humid conditions. In other European countries *Oxidus gracilis* is also common in greenhouses, city parks and gardens (Stoev and Korsós 2010). Other species, like the North African *Cylindroiulus truncorum* and the European species *Boreoiulus tenuis*, *Choneiulus palmatus* and *Nopoiulus kochii*, are restricted to anthropogenic, synanthropic habitats and greenhouses. *Cylindroiulus vulnerarius* is hitherto known only from a garden in Waltrop, North Rhine-Westphalia (Decker and Hannig 2011) suggesting that this species can also survive in urban habitats. Although currently there are no outdoor records of *Mesoiulus gridellii* known from Germany, this species, originally reported from Italy, is known from several urban sites in adjacent Austria (Gruber 2002) and could also inhabit similar biotopes (e.g. gardens, parks or subterranean passageways) in Germany.

## Supplementary Material

Supplementary material 1 Occurrence data of Diplopoda and Chilopoda from German greenhouses Data type: Microsoft Excel spreadsheet
Brief description: Occurrence data of Diplopoda and Chilopoda from German greenhouses, including the name of the federal state, city, location, site, coordinates, micro-habitat, sampling date, collector, sampled specimens, collection, notes and literature.
File: oo_6060.xls Peter Decker & Hans Reip

Supplementary material 2 Distribution of German greenhouse myriapods across cities Data type: Microsoft Excel spreadsheet
Brief description: The dataset provides a complete list of greenhouse myriapods and their distribution across the cities.
File: oo_6195.xls Peter Decker, Hans Reip & Karin Voigtländer

XML Treatment for Chilopoda

XML Treatment for Lithobiomorpha

XML Treatment for Henicopidae

XML Treatment for Lamyctes coeculus

XML Treatment for Lithobiidae

XML Treatment for Lithobius aeruginosus

XML Treatment for Lithobius crassipes

XML Treatment for Lithobius forficatus

XML Treatment for Lithobius lapidicola

XML Treatment for Lithobius melanops

XML Treatment for Lithobius microps

XML Treatment for Lithobius mutabilis

XML Treatment for Scolopendromorpha

XML Treatment for Cryptoptidae

XML Treatment for Cryptops hortensis

XML Treatment for Cryptops doriae

XML Treatment for Geophilomorpha

XML Treatment for Dignathodontidae

XML Treatment for Henia vesuviana

XML Treatment for Geophilidae

XML Treatment for Geophilus flavus

XML Treatment for Geophilus electricus

XML Treatment for Pachymerium ferrugineum

XML Treatment for Himantariidae

XML Treatment for Haplophilus subterraneus

XML Treatment for Mecistocephalidae

XML Treatment for Mecistocephalus maxillaris

XML Treatment for Tygarrup javanicus

XML Treatment for Schendylidae

XML Treatment for Pectiniunguis pauperatus

XML Treatment for Diplopoda

XML Treatment for Julida

XML Treatment for Blaniulidae

XML Treatment for Archiboreoiulus pallidus

XML Treatment for Blaniulus guttulatus

XML Treatment for Boreoiulus tenuis

XML Treatment for Choneiulus palmatus

XML Treatment for Nopoiulus kochii

XML Treatment for Proteroiulus fuscus

XML Treatment for Julidae

XML Treatment for Brachyiulus pusillus

XML Treatment for Cylindroiulus britannicus

XML Treatment for Cylindroiulus caeruleocinctus

XML Treatment for Cylindroiulus latestriatus

XML Treatment for Cylindroiulus punctatus

XML Treatment for Cylindroiulus truncorum

XML Treatment for Cylindroiulus vulnerarius

XML Treatment for Kryphioiulus occultus

XML Treatment for Mesoiulus gridellii

XML Treatment for Ommatoiulus sabulosus

XML Treatment for Unciger foetidus

XML Treatment for Spirobolida

XML Treatment for Rhinocricidae

XML Treatment for Anadenobolus monilicornis

XML Treatment for Spirobolellidae

XML Treatment for Paraspirobolus lucifugus

XML Treatment for Trigoniulidae

XML Treatment for Leptogoniulus sorornus

XML Treatment for Spirostreptida

XML Treatment for Epinannolenidae

XML Treatment for Epinannolene cf. trinidadensis

XML Treatment for Epinannolene sp.

XML Treatment for Polyzoniida

XML Treatment for Siphonotidae

XML Treatment for Rhinotus purpureus

XML Treatment for Polydesmida

XML Treatment for Haplodesmidae

XML Treatment for Cylindrodesmus hirsutus

XML Treatment for Prosopodesmus jacobsoni

XML Treatment for Oniscodesmidae

XML Treatment for Amphitomeus attemsi

XML Treatment for Paradoxosomatidae

XML Treatment for Oxidus gracilis

XML Treatment for Strongylosoma stigmatosum

XML Treatment for Polydesmidae

XML Treatment for Brachydesmus superus

XML Treatment for Polydesmus angustus

XML Treatment for Polydesmus complanatus

XML Treatment for Polydesmus inconstans

XML Treatment for Pyrgodesmidae

XML Treatment for Poratia digitata

XML Treatment for Poratia obliterata

XML Treatment for Chordeumatida

XML Treatment for Chordeumatidae

XML Treatment for Melogona voigtii

## Figures and Tables

**Table 1.** List of all investigated greenhouses in Germany.

City	Name of locality	Literature	(Re-)Investigated in this study
Bayreuth	Bayreuth Ecological Botanical Garden		×
Berlin	Berlin Old Botanical Garden	Eichler 1952	
	Berlin-Dahlem Botanical Garden	Boettger 1929, Eichler 1952, Golovatch et al. 2001, Schubart 1934, Schubart 1929b	×
	Berlin Zoological Garden		×
	(Cemetary) nurseries, without exact locality	Schubart 1929b, Schubart 1957	
	Horticulture company in Berlin-Zehlendorf	Boettger 1929	
Bochum	Bochum Botanical Garden		×
Bonn	Bonn Botanical Garden	Decker and Hannig 2011	×
	Hothouse of Mr. Biesing	Verhoeff 1891	
Darmstadt	Darmstadt Botanical Garden		×
Dresden	Dresden Botanical Garden	Richter 1967	×
Düsseldorf	Aquazoo-Löbbecke Museum	Adis et al. 2000	
Duisburg	Hothouse, without exact locality		×
Frankfurt am Main	Palm Garden	Heußler 2009	×
Gießen	Gießen Botanical Garden	Läffert 1984	
Halle	Halle Botanical Garden	Schubart 1934	×
Hamburg	Hamburg Botanical Garden	Jeekel 1964a, Silvestri 1907	×
	Hothouses in Hamburg-Wandsbeck	Hahmann 1929a, Hahmann 1929b	
	Nursery, without exact locality	Latzel 1895	
	Tannery, without exact locality	Latzel 1895	
	Greenhouse, without exact locality	Latzel 1895	
Hannover	Hothouse, without exact locality	Schubart 1934	
Jena	Jena Botanical Garden		×
Kamen	Hothouse, without exact locality	Verhoeff 1934	
Karlsruhe	Karlsruhe Botanical Garden		×
Kiel	Kiel Botanical Garden	Adis et al. 2000, Wilck 2000	
	Greenhouse, without exact locality		×
Köln/Cologne	Cologne Botanical Garden	Decker and Hannig 2011	×
	Zoological Institut at Weyertal	Thiele 1968	
Konstanz	Mainau, Butterfly house		×
Leipzig	Leipzig Botanical Garden		×
	Zoological Garden Leipzig, Gondwanaland		×
	Nursery garden and horticulture		×
Lübeck	Palm house	Schubart 1925	
Mainz	Mainz Botanical Garden		×
	City Park		×
Magdeburg	Gruson-Greenhouses		×
Marburg	Marburg Botanical Garden		×
Marlow	Ornithological Park Marlow		×
München/Munich	München-Nymphenburg Botanical Garden	Boettger 1929	×
Oldenburg	Oldenburg Botanical Garden		×
Potsdam	Potsdam Botanical Garden		×
	Terrace nursery Park Sanssouce	Schubart 1929b	×
	Biosphere Potsdam		×
	Horticulture company in Potsdam-Neubabelsberg	Schubart 1957	
Rostock	Rostock Botanical Garden	Schubart 1929a	
Tharandt	Tharandt Botanical Forest Garden		×
Trier	Greenhouse, without exact locality	Schnur 1857	
